# A redox sensor in protein kinase A regulatory subunit Iα regulates vasodilation and protects against hypertension

**DOI:** 10.1016/j.redox.2026.104136

**Published:** 2026-03-23

**Authors:** Olena Rudyk, Alice Braga, Naomi Wheatcroft, Mattia Bonzanni, Oleksandra Prysyazhna, Michelle N.A. Korneh, Hannah L.H. Green, Yang Zhou, Min Zhang, Alexander V. Gourine, Philip Eaton

**Affiliations:** a School of Cardiovascular and Metabolic Medicine & Sciences, Faculty of Life Sciences & Medicine, King's College London, London, UK; b Centre for Cardiovascular and Metabolic Neuroscience, Department of Neuroscience, Physiology and Pharmacology, University College London, London, UK; c Department of Pediatrics, Weill Cornell Medical College, New York, USA; d William Harvey Research Institute, Queen Mary University of London, UK

**Keywords:** Redox sensing and signaling, PKARIα, Hypertension, Angiotensin II, Oxidant stress

## Abstract

The regulatory RIα subunit of type I protein kinase A (PKARIα) can be oxidized to an interprotein disulfide homodimer, linking cellular oxidant signals with downstream phosphorylation-mediated physiological regulation. Although PKA and oxidants each have well-established roles in vasodilation and blood pressure lowering, whether the oxidation of RIα to the disulfide state regulates these physiological processes is unknown. Here, we demonstrate that PKARIα contains a thiol redox sensor, the oxidation state of which is dynamic and modulates systemic arterial blood pressure by regulating oxidant-induced vasodilation. Mesenteric or carotid arteries, penetrating cerebrovascular arterioles or aortae isolated from ‘redox-dead’ Cys17Ser PKARIα knock-in mice that are resistant to disulfide-dimer formation demonstrate enhanced responses to vasoconstrictors and impaired vasodilation induced by oxidants, but not nitric oxide or cAMP elevation, compared to wild-type preparations. Although administration of angiotensin II increased vasoconstriction, this pro-oxidant vasopressor hormone concomitantly increased vasodilatory disulfide-PKARIα and limited the development of hypertension and pressure-induced cardiac hypertrophy in a mouse model. These observations indicate a role for disulfide-PKARIα in vasodilation; however, the knock-in mice were normotensive with blood pressure similar to that of wild-type controls. Heart rate variability analysis showed that the knock-in mice had a lower low-to-high frequency ratio than wild-type mice, pointing to reduced sympathetic activity in the ‘redox-dead’ transgenic mice. This was corroborated biochemically by lower norepinephrine levels in renal and cardiac tissues of the knock-in mice. Reduced sympathetic activity would explain why the PKARIα knock-in animals are not hypertensive despite their arteries having enhanced constrictor responses to vasopressors. Together, the data obtained in this study provide strong evidence that oxidation of PKARIα couples to arterial dilation and mitigates blood pressure increases, highlighting this kinase as a potential new target for thiol-based therapeutics to treat systemic hypertension.

## 1 Introduction

Systemic arterial hypertension affects over one billion individuals globally and remains the leading cause of cardiovascular morbidity and mortality. It poses a well-recognized risk factor for stroke [1], myocardial infarction [2,3], heart failure [2], peripheral artery disease [4,5], chronic kidney disease [6], aortic aneurysm [7] and diabetes mellitus [8]. Beyond these life-threatening complications, high blood pressure may substantially reduce the quality of life in affected individuals. However, it often causes no symptoms, leading to fatal, undetected, and long-term health consequences. At the molecular level, hypertension is characterized by an imbalance between physiological redox signaling and pathological oxidative stress across multiple organ systems, including the vasculature, heart, kidneys, and immune cells [9]. Identifying novel oxidant-mediated sensing and signaling mechanisms that regulate systemic blood pressure may facilitate the development of more effective treatments for this chronic condition.

3′, 5′-cyclic adenosine monophosphate (cAMP) dependent protein kinase A (PKA) is abundantly expressed in the cardiovascular system, where it plays several regulatory roles [10,11]. Once activated by beta-adrenergic stimulation, canonical PKA signaling orchestrates fight-or-flight stress responses, leading to combined inotropic, chronotropic and lusitropic cardiac effects [12,13]. In addition, PKA plays an important role in skeletal muscle and digestive tract function and in regulating learning and memory processes in the brain [14,15]. PKA is expressed in many other cell types where it controls lipid and carbohydrate metabolism [16], gene transcription, angiogenesis, cellular proliferation [17], and immune function [18] by catalyzing the phosphorylation of PKA-dependent substrates [10,19]. Notably, PKA plays a crucial regulatory role in vascular function, growth, and permeability, as well as in fluid homeostasis and in the secretion of renin by the kidneys to regulate blood pressure [10,20,21]. The latter is also determined by cardiac output and the resistance provided by the peripheral circulation, such that alterations in the contractile properties of the heart or arteries can lead to high blood pressure.

**Fig. 1:**
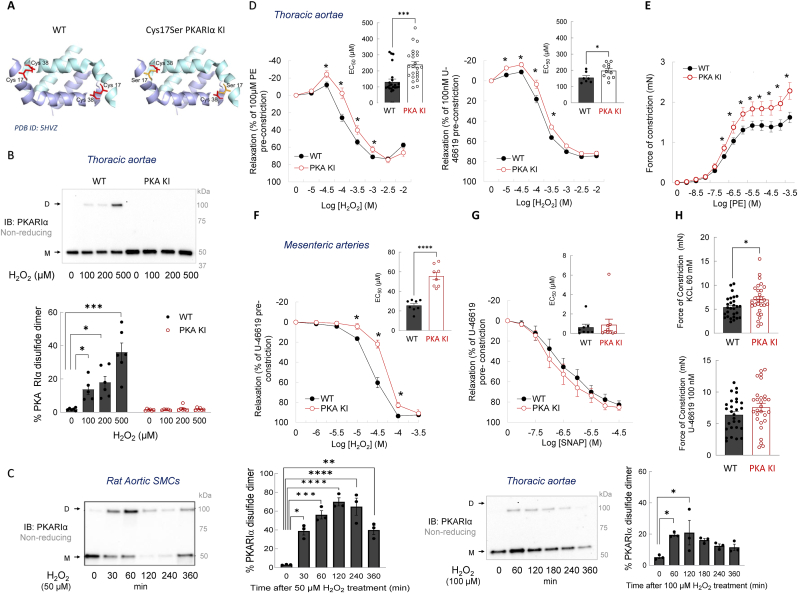
Comparative phenotyping of *ex vivo* arteries isolated from wild-type or ‘redox-dead’ Cys17Ser PKARIα knock-in mice. **A.** Structural representation of PKARIα docking domain (PDB ID: 5HVZ, facilitated by PyMOL) indicating positions of Cys17 and Cys38 that form a homodimeric disulfide bond. Left – PKARIα wild type (WT) protein; right – Cys17Ser PKARIα (PKA KI) mutant protein. **B.** Disulfide-PKARIα protein expression in thoracic aortae rings of WT or PKA KI mice subjected either to a vehicle or 100, 200 and 500 μM H_2_O_2_ treatment for 30 min (n ≥ 5). **C.** Disulfide-PKARIα protein expression in rat aortic smooth muscle cells subjected to 50 μM H_2_O_2_ treatment for 30 - 360 min (left) or thoracic aortae rings of WT mice subjected to 100 μM H_2_O_2_ treatment for 60 - 360 min (right) (n = 3). **D.** H_2_O_2_-induced dose-dependent relaxation of aortic rings (pre-constricted either with PE or U-46619), from WT or PKA KI mice (n ≥ 5). **E.** Dose-dependent constriction of aortic rings from WT or PKA KI mice in response to PE (n ≥ 5). **F.** Dose-dependent relaxation of mesenteric arteries from WT or PKA KI mice in response to H_2_O_2_ (n ≥ 5). **G.** Dose-dependent relaxation of mesenteric arteries from WT or PKA KI mice in response to SNAP (n ≥ 5). **H.** Constriction of mesenteric arteries from WT or PKA KI mice in response to a single dose of KCl (top) or U-46619 (bottom) (n ≥ 5)*. ∗P< 0.05, ∗∗P< 0.01, ∗∗∗P< 0.001, ∗∗∗∗P< 0.0001 vs. vehicle or respective WT. WT, wild type; PKA KI, Cys17Ser PKARIα knock-in mice; M, PKARIα monomer; D, PKARIα dimer; SMCs, smooth muscle cells; H*_*2*_*O*_*2*_*, hydrogen peroxide, acts a vasodilator; PE, phenylephrine, acts as α1-adrenoceptor agonist; SNAP, S-nitroso-N-acetyl-d,**l**-penicillamine, acts as a donor of nitric oxide; U-46619, stable synthetic analogue of the prostaglandin PGH2, acts as a thromboxane receptor agonist; KCl, potassium chloride, acts as activation of voltage-operated calcium channels.*

PKA exists as type I and type II isozymes, which are distinguished by their different RI or RII regulatory subunits that interact with and inhibit the catalytic subunits [11,22]. Each specific subtype is therefore a tetramer composed of two catalytic and two regulatory subunits, defined by the R type in the holoenzyme. The canonical mode of PKA activation involves a binding of an activating ligand, namely the cAMP, generated because of stimulatory (Gs) protein-coupled receptor and membrane-bound adenylyl cyclase activation. cAMP binds to each of the two regulatory subunits, leading to partial dissociation [23] and activation of the two catalytic subunits present in the tetrameric holoenzyme complex [11]. Once dissociated from the tetrameric complex, PKA catalytic subunits then phosphorylate serine or threonine residues on substrate proteins to regulate their functional activities and physiological processes [10,11,22]. In addition, due to alternative splicing, α and β isoforms exist for each regulatory subunit [10]. The former are dimeric by virtue of an N-terminal amphipathic leucine zipper in which the monomers are aligned antiparallel to each other. In the RIα isoform, each Cys17 residue within a dimer pair directly faces the corresponding Cys38 residues on the opposite chains [24]. When cellular oxidants are increased, these vicinal cysteine thiols in RIα form an interprotein disulfide (Fig. 1A), an oxidation product associated with non-canonical regulation of PKA, characterized by subcellular translocation and activation of the kinase [[25], [26], [27]]. While the PKARIα disulfides do not seem to directly affect the canonical cAMP-stimulated kinase activity, they likely modulate subcellular localization *via* altered binding to A-kinase anchoring proteins [10,28]. In hearts undergoing ischemia-reperfusion, oxidation of PKARIα is shown to redistribute the PKA holoenzyme to lysosomes in wild-type preparations. This protective mechanism, which modulates calcium (Ca^2+^) signaling and reduces ischemic damage, is impaired in ‘redox-dead’ Cys17Ser PKARIα knock-in (PKA KI) mice that are unable to sense and transduce signaling through disulfide-PKARIα [29]. Despite recent efforts to understand the functional mechanistic role of the PKARIα-disulfides in the heart [[29], [30], [31]], its role in the vascular system remains largely unexplored.

It is well established that oxidant molecules, which can induce disulfides in proteins such as PKARIα, dilate arteries independently of the nitric oxide (NO) pathway [[32], [33], [34]]. Indeed, resistance arteries and arterioles, whose tone is a principal determinant of blood pressure, are more susceptible to oxidant-induced vasodilation than large conduit blood vessels [35]. Given that both PKA and oxidants play well-established roles in vasodilation and blood pressure reduction [32,33,36,37], thereby promoting tissue perfusion, we investigated whether non-canonical modulation of PKARIα activity by redox switching of RIα between reduced and disulfide states regulates these processes across multiple vascular beds *ex vivo*. We further examined whether this mechanism translates into a physiological role for this non-canonical PKARIα activation in a chronic *in vivo* model of systemic hypertension.

It was found that conduit and resistance arteries isolated from PKA KI mice, which are unable to form disulfides in response to endogenous or exogenous oxidants, exhibited impaired oxidant-induced vasodilation. Treatment with the oxidant-generating vasoconstrictor angiotensin II (AngII), which concomitantly increases vasodilatory disulfide-PKARIα, resulted in exaggerated vasoconstriction of arteries and arterioles, more severe systemic hypertension and pressure-induced cardiac hypertrophy in the transgenic animals. These findings demonstrate that PKARIα disulfide dimer formation serves as a protective vascular mechanism that facilitates arterial relaxation and counteracts vasoconstriction, particularly under conditions associated with increased oxidant production such as chronic systemic hypertension. Collectively, our data identify the PKARIα redox sensor as a previously unrecognized non-canonical regulatory mechanism of oxidant-mediated vasodilation and highlight its therapeutic potential as a target for thiol-based strategies to lower blood pressure. Some results from this study have been reported previously in abstract form [38].

## 2 Materials and methods

### 2.1 Animals

All animal procedures were performed in accordance with the Home Office Guidance on the Operation of the Animals (Scientific Procedures) Act 1986 in the United Kingdom and were approved by the King's College Animal Welfare and Ethical Review Body. Mice constitutively expressing PKARIα Cys17Ser were produced on a pure C57BL/6 background by Taconic Artemis as described [39]. Nox2−/y (Nox2-null) mice were obtained from Jackson Laboratories [40,41]. Nox4-null mice were generated by targeted deletion of the translation initiation site and exons 1 and 2 of the gene by Genoway, as described [42,43]. Matched wild-type (WT) littermates were used as controls with the same genetic background (C57BL/6J). All animals were bred on-site, had *ad libitum* access to standard chow and water and were kept in specific pathogen-free conditions under a 12-h day/night cycle at 20 °C and 60% humidity. Cages, water, and food were changed every 7-10 days for all animals. Age- and body weight-matched WT or PKA KI, Nox2-null or Nox4-null male offspring were employed. Mice aged 12-16 weeks (young) were used in most experiments. In some cases, mice aged 12-16 months (mid-aged) were also employed. To avoid confounding by circadian rhythms, all experiments within a study were performed at approximately the same time of day, in a randomized and blinded manner.

### 2.2 Small-vessel myography

Thoracic or abdominal aortae vascular rings, left and right common carotid arteries and second-order mesenteric arteries were mounted for isometric tension recordings in a tension myograph (Danish Myo Technology, DMT), stretched to the optimal pretension condition with a DMT normalization module, and bathed in Krebs solution at 37.0 °C with a 95% O_2_ and 5% CO_2_ environment, as before [33,44,45]. Vessel size of the left and right common carotid arteries was calculated through LabChart DMT normalization module and expressed as internal circumference. Vascular responses to hydrogen peroxide (H_2_O_2_, 10 nM to 10 mM), *S*-nitroso-*N*-acetyl-d,l-penicillamine (SNAP, 30 nM to 300 μM), S-nitroprusside (SNP, 0,1 nM to 100 μM), phenylephrine (PE, 1 nM to 300 μM), U-46619 (3 nM to 10 μM), acetylcholine (Ach, 10 nM to 100 mM), 5′-*N*-ethylcarboxamidoadenosine (NECA, 10 nM to 1 mM) or AngII (100 nM to 1 μM) were assessed with endothelium-intact isolated mouse vessels. The plateau responses of precontracted (by the α-adrenoceptor agonist phenylephrine or the thromboxane mimetic U-46619) vessels to the potentially vasodilatory drugs were determined. Constricting dose-responses to phenylephrine or U-46619, acute vasoconstriction to potassium chloride (KCl) or a single dose of AngII were assessed with endothelium-intact isolated mouse vessels.

### 2.3 Assessment of blood flow

Basal arterial antegrade flow was measured non-invasively in carotid arteries of anesthetized mice using the Vevo F2 system (VisualSonics, Toronto, ON, Canada) equipped with a UHF46X transducer. Mice were placed in the supine position under volatile anesthesia with 2% (vol/vol) isoflurane (Centaur Services) delivered in 1 L of oxygen *per* minute. All limbs were taped to ECG electrodes for continuous heart rate monitoring, and core body temperature was maintained at 37.0 ± 0.5 °C using a rectal temperature probe. High-resolution pulsed-wave Doppler in combination with B-mode imaging was applied to assess carotid flow parameters at the left or right common carotid artery, proximal to the bifurcation. Because of the transducer orientation, the Doppler probe recorded velocities as negative, but they are reported here as positive absolute values. Cardiac function was assessed by B-mode and M-mode images obtained at the level of the papillary muscles. Data were analyzed offline with VevoLab software (VisualSonics, Toronto, ON, Canada). Cardiac index was calculated by normalizing cardiac output to each mouse's body mass.

### 2.4 Two-photon imaging of cerebral vasculature *in vivo*

#### 2.4.1 Animal preparation

Mice were anesthetized with an intravenous injection of fentanyl (0.05 mg/kg), midazolam (5 mg/kg) and medetomidine (0.5 mg/kg). Room air supplemented with oxygen (∼30% O_2_) was supplied through a nose mask, and mice breathed unaided throughout the experiment. Body temperature was maintained at 37.0 ± 0.5 °C. The head of the animal was secured in a stereotaxic frame, and a small circular craniotomy (∼3 mm^2^) was made in the skull above the somatosensory cortex. Cortical cells were loaded with a Ca^2+^-sensitive dye Oregon Green BAPTA 1 a.m. (OGB). OGB was first dissolved in DMSO and Pluronic F127 (20%). The solution containing OGB (1 mM) in artificial cerebrospinal fluid (aCSF; 124 mM NaCl, 3 mM KCl, 2 mM CaCl_2_, 26 mM NaHCO_3_, 1.25 mM NaH_2_PO_4_, 1 mM MgSO_4_, 10 mM d-glucose saturated with 95% O_2_/5% CO_2_, pH 7.4) was delivered by microinjection *via* a glass micropipette at 2-4 sites within the targeted area of the cortex. The exposed surface of the brain was then covered with agarose (1%) and protected with a glass coverslip secured to the skull with a headplate and acrylic dental cement. Intravascular fluorescent dye Texas Red (15 mg/kg; MW 70,000, ThermoFisher) or Fluorescein isothiocyanate (FITC)-Dextran (15 mg/kg; MW 40,000, Sigma) were administered intravenously.

Vascular and cellular responses in the cortex were recorded using an Olympus FV1000 microscope (Olympus), equipped with MaiTai HP DeepSee laser (Spectra-Physics). A 25x water-immersion objective (XLPlan N, NA 1.05; Olympus) was used. Fluorophores were excited in two-photon XYZ-t mode at 800 nm. Penetrating cortical arterioles were identified by their anatomical characteristics, including branching pattern and lumen diameter and by fluorescence of Texas Red or FITC-Dextran. Perivascular astrocytes were identified by their endfeet ensheathing the vessel wall. Laser power was kept to a minimum to reduce phototoxicity. Time-lapse recordings were made for up to 11 min with a period of hypoxia (10% O_2_ in the inspired gas, balanced with N_2_) lasting for 1 min, as described previously [46]. For evaluation of AngII responses, 50 μg/kg of AngII was injected intravenously in a volume of 50 μl and vessel responses were recorded for a period of up to 10 min.

#### 2.4.2 Sen's slope estimator

Sen's slope estimator was used to quantify the rate of change over time upon AngII injections. The method calculates the median slope from all possible pairwise combinations of data points in the time series. Specifically, the slope between each pair of points (i,j), with i < j, is computed as:

$${Slope}_{ij}=\frac{x_{j}-x_{i}}{t_{j}-t_{i}}$$

where x is the observed value and t is the time. All slopes are then aggregated, and the median is taken as the estimated trend, representing the magnitude of change per unit time. Custom-made MATLAB code has been used for the analysis. For the analysis, one point every five has been used.

#### 2.4.3 Vessel imaging analysis

Videos of vascular recordings were processed in ImageJ, single frames were isolated, and a maximum Z-projection was computed. On the Z-projection image, a region of interest (ROI) was selected, saved, and used to crop the individual frames, which were then saved for further analysis. By performing the Z-projection and selecting a stable ROI, we accounted for movements of the vessel during dilations and contractions. Custom-made MATLAB code, adapted from established image analysis protocols [47,48], was used to identify the vessel boundary and extract morphometric parameters, including cross-sectional area, major axis, minor axis, and equivalent diameter. Equivalent diameter, as a proxy of vessel caliber, was used for further analysis. Delta was calculated as the difference in diameter between the peak and the baseline for each experimental event. The kinetics (rise and decay slopes) were determined by manually fitting a linear regression to the rising and decaying phases, respectively, during their linear segments.

### 2.5 Blood pressure, stress responses and heart rate variability measurements

Blood pressure, heart rate and locomotor activity were assessed by telemetry in freely moving mice as described before [33,44,45]. Briefly, mice were anesthetized with 2% (vol/vol) isoflurane in 0.5 L of oxygen *per* minute with pre- and postoperative analgesia (buprenorphine, 0.1 mg/kg, Abbot Laboratories), and a TA11PA-C10 probe catheter (Data Science International, Inc) was implanted into the aortic arch *via* the left carotid artery. Following a 7-10 days’ recovery, mice were placed above the telemetric receivers, and the blood pressure was recorded by scheduled sampling over the weekends. The stress response was assessed by placing mice in a new cage with fresh bedding and no enrichment. Frequency-domain short-term heart rate variability (HRV) parameters were analyzed, as before [49,50]. A 1-h blood pressure record was obtained during quiet time in the early morning (usually, Mondays, between 8 a.m. and 10 a.m.), and the analyses were performed using the HRV module of Chart 7.0 software (ADInstruments, Colorado Springs, CO). Integrated boundaries for the spectral bands were set at 0.4-1.5 Hz for the low-frequency (LF) component and 1.5-5 Hz for the high-frequency (HF) component.

### 2.6 Chronic infusion of angiotensin II

In some experiments, mice were subcutaneously implanted with Alzet osmotic minipumps (2004, Charles River, UK) for continuous AngII (Sigma-Aldrich, UK) delivery at 1.2 mg/kg/day for 21 days. Pumps were primed overnight at 37 °C before implantation surgery, which was performed in anesthetized mice receiving 2% isoflurane in 0.5 L of oxygen *per* minute, with pre- and postoperative analgesia (buprenorphine, 0.1 mg/kg).

Cardiac function after chronic AngII infusion was assessed by non-invasive cardiovascular imaging. Mice were anesthetized with 2% isoflurane in 0.5 L of oxygen *per* minute and examined using a Vevo770 echocardiography system (VisualSonics, Toronto, ON, Canada) with an RMV-707B transducer operating at 30 MHz. The core body temperature was maintained at 37.0 ± 0.5 °C with a feedback-regulated body temperature probe. High-resolution, two-dimensional B-mode and M-mode images were obtained at the level of the papillary muscles and further analyzed offline with VevoLab Software (VisualSonics) as before [45,51]. In some experiments, after completion of echocardiographic imaging, mice were euthanized, and blood was rapidly sampled from the inferior vena cava and immediately analyzed using an iSTAT blood biochemistry analyzer with EC8+ iSTAT cartridges (Abaxis, UK), as described previously [45].

### 2.7 Catecholamine assay

Catecholamine assays were performed by the Vanderbilt University Medical Center Hormone Assay and Analytical Services Core. Norepinephrine was measured by HPLC with electrochemical detection. Samples were absorbed onto alumina at a pH of 8.6, eluted with dilute perchloric acid, and auto-injected onto a C18 reversed-phase column. An internal standard (dehydroxylbenzylamine) is included with each extraction to monitor recovery and standard curves for both epinephrine and norepinephrine are run. Results were quantitated through a chromatography data station [52,53].

### 2.8 Cultured cells and immunoblotting

Primary vascular smooth muscle cells (SMCs) were isolated from male Wistar rat aortas as before [54]. Experiments were performed on cells from passage 6 to 12. Rat aortic SMCs were cultured in DMEM, supplemented with 10% fetal bovine serum and 1% penicillin/streptomycin, in 12-well plates in 37 °C with a 95% O_2_–5% CO_2_ environment. Once confluent, cells were treated with AngII (Sigma-Aldrich, UK), H_2_O_2_ (Sigma-Aldrich, UK) or vehicle, and lysed in sample buffer with the addition of 100 mmol/L maleimide for further analysis. Samples were then resolved on a standard SDS-PAGE gel, immunoblotted, and probed with an antibody for PKARI. Immunoblotting for PKARIα disulfide dimer was performed as described previously [25,39]. Maleimide (100 mmol/L) was used in preparation buffers to alkylate free thiols and prevent thiol disulfide exchange. Antibodies used in these studies included PKARI (BD Biosciences or Cell Signaling Technologies). Horseradish peroxidase–linked secondary antibody (Cell Signaling Technologies) and Pierce™ ECL Western Blotting Substrate (ThermoFisher Scientific) were used. The proportion of reduced to oxidized PKARIα was then quantified with GelPro Analyzer or Image J.

### 2.9 Treatment of isolated vessels

Mice were euthanized by pentobarbital overdose, and thoracic aortae were isolated and cleaned from surrounding tissues and fat in an ice-cold Krebs solution. Aortae rings (5 mm) were incubated with 100 to 500 μM H_2_O_2_ or 10 nM to 10 μM AngII in Krebs at 37 °C with a 95% O_2_/5% CO_2_ environment as times indicated (i.e., for 30 min or up to 6 h), frozen and homogenized in liquid nitrogen with sample buffer with the addition of 100 mmol/L maleimide for further analysis. Samples were then resolved on a standard SDS-PAGE gel, immunoblotted, and probed with an antibody for PKARI. The proportion of reduced to oxidized PKARIα was then quantified with GelPro Analyzer or Image J.

### 2.10 Statistical analysis

All results are presented as individual values and means ± SEM. Differences between groups were assessed by ANOVA followed by post-hoc tests. Differences were considered significant at the 95% confidence level. Relevant analyses were performed with GraphPad Prism 8.4.2. Statistical analysis of cerebral vessel imaging data (delta, kinetics) was conducted using nested *t*-test. Dependencies in the structure of the data (repetitive nature: hypoxia stimulus repeated for the same vessel; more than one vessel recorded from the same mouse) have been accounted for in the nested model.

### 2.11 Code availability

The code used for vessel analysis is available on Open Science Framework (OSF) at the following link: https://osf.io/g7b45.

## 3 Results

### 3.1 PKARIα disulfide dimerization at Cys17-Cys38 mediates vasorelaxation

To test whether PKARIα undergoes oxidative disulfide dimer formation in vascular cells and intact arteries, rat aortic SMCs or mouse thoracic aortae rings were exposed to increasing concentrations of H_2_O_2_ for 30 min. Non-reducing immunoblot analysis revealed that PKARIα was largely, but not fully, reduced under basal conditions in both SMCs and intact arteries, and as anticipated, exposure to H_2_O_2_ promoted increased oxidation to the PKARIα disulfide dimer (Suppl Fig. 1A and 1B, C). Time-course experiments demonstrated that disulfide-PKARIα formation was sustained for up to 4 h in rat aortic SMCs and for up to 3 h in WT aortic rings following H_2_O_2_ exposure (50 and 100 μM, respectively) (Fig. 1C). However, prolonged treatment led to a gradual decline in disulfide dimer levels (Fig. 1C), suggesting the dynamic and reversible nature of this redox modification and its regulation by intracellular reducing systems.

To determine if the disulfide-PKARIα mediates H_2_O_2_-induced vasodilation, vascular responses were compared between arteries from WT and PKA KI mice. The PKA KI mice were genetically engineered to have a constitutive mutation in which cysteine (Cys) 17 was replaced with a serine (Ser) (Fig. 1A), meaning the cysteine thiol side chain is replaced by a hydroxyl group - providing a conservative single atom alteration that is sufficient to remove the oxidant sensing and signaling mediated by disulfide formation in PKARI. As anticipated, arteries expressing Cys17Ser RIα showed no disulfide either basally or in response to H_2_O_2_ (Fig. 1B), confirming effective abrogation of PKARIα redox signaling in the transgenics. Thoracic aortic rings were pre-constricted with either the α_1_-adrenoceptor agonist phenylephrine or the thromboxane mimetic U-46619 and subsequently exposed to cumulative increasing concentrations of H_2_O_2_. H_2_O_2_ induced concentration-dependent vasodilation in WT arteries; however, this response was significantly attenuated in aortae from PKA KI mice. This impairment was reflected by a rightward shift in the concentration-response curve and a corresponding increase in EC_50_ values in preparations lacking the PKARIα redox sensor (Fig. 1D). These data provide robust evidence that disulfide bond formation in PKARIα plays a causal role in arterial dilation in response to oxidants. Consistent with the loss of an endogenous vasodilatory mechanism, concentration-dependent constriction to phenylephrine was significantly greater in arteries from PKA KI mice compared with WT controls (Fig. 1E). Thromboxane agonist-induced concentration-dependent constriction was largely similar in arteries (Suppl. Fig. 1B); however, it should be noted that many of the U-46619 concentrations employed produced supraphysiological levels of contraction. As such, maximal force generation in response to U-46619 (i.e., ∼5-fold greater than that induced by phenylephrine) likely limited the capacity to detect genotype-dependent differences at higher concentrations. Importantly, at lower, physiologically relevant concentrations (∼30 nM), U-46619 elicited significantly greater constriction in arteries from PKA KI mice compared with WT vessels, recapitulating the hypercontractile phenotype observed with phenylephrine.

Although aortae from PKA KI mice exhibited impaired oxidant-induced dilation, the physiological relevance of these observations to blood pressure control is questionable, as changes in the tone of the largest conduit arteries are unlikely to exert a major influence on systemic blood pressure regulation. Because arterial pressure is primarily determined by the caliber of small resistance vessels, the vasomotor responses of mesenteric arteries from WT and PKA KI mice were examined next. Assessing these vessels is particularly relevant physiologically, as resistance arteries are highly sensitive to oxidant-induced vasodilation [35] and hypertension is primarily caused by their dysfunction [55]. Mesenteric arteries isolated from PKA KI mice and constricted with U-46619 displayed significantly attenuated dilation upon exposure to H_2_O_2_ compared to those from WT (Fig. 1F). In contrast, concentration-dependent relaxation induced by the nitric oxide donor SNAP was similar between genotypes (Fig. 1G), consistent with the inability of SNAP to induce PKARIα disulfide formation at the concentrations examined [56]. Interestingly, mesenteric arteries from PKA KI mice generated greater force in response to depolarization with potassium chloride (60 mM) than WT controls, whereas constriction to U-46619 (100 nM) did not reach statistical significance (Fig. 1H). Taken together, the differential vasomotor responses observed in arteries lacking disulfide-PKARIα sensing and signaling provide strong evidence that PKARIα-disulfide formation causally contributes to aortic dilation in response to oxidants.

### 3.2 PKARIα redox sensor contributes to brain blood flow regulation

**Fig. 2:**
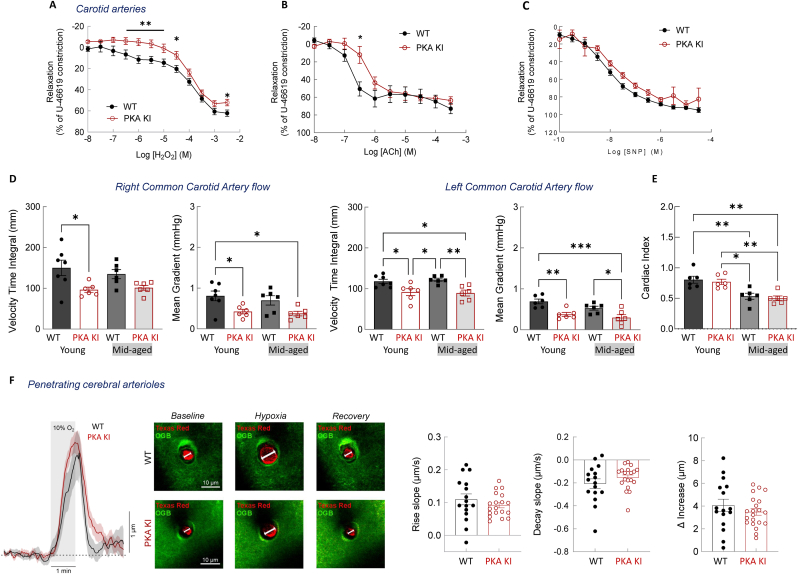
Comparison of *ex vivo* and *in vivo* responses in carotid arteries and penetrating cerebral arterioles of wild-type or ‘redox-dead’ Cys17Ser PKARIα knock-in mice. **A-C.** H_2_O_2_-induced (**A**), Ach-induced (**B**) and SNP-induced (**C**) dose-dependent relaxation of carotid arteries (pre-constricted with U-46619), from WT or PKAKI mice (n ≥ 6). **D-E.** Carotid arterial flow parameters (**D**) assessed by ultrasound Doppler, and cardiac index (**E**) assessed by M-mode ultrasound imaging in anesthetized WT or PKA KI mice (n ≥ 6). **F.** Responses of penetrating cerebral arterioles to acute systemic hypoxia (10% O_2_ in the inspired air) in anesthetized WT or PKA KI mice (n ≥ 10). *∗P< 0.05, ∗∗P< 0.01, ∗∗∗P< 0.001 vs. respective WT. WT, wild type; PKA KI, Cys17Ser PKARIα knock-in mice; H*_*2*_*O*_*2*_*, hydrogen peroxide, acts a vasodilator; Ach, acetylcholine, muscarinic receptors agonist, acts as a vasodilator; SNP, S-nitroprusside, acts as a donor of nitric oxide/vasodilator; U-46619, stable synthetic analogue of the prostaglandin PGH2, acts as a thromboxane receptor agonist; OGB, Oregon Green 488 BAPTA-1, cell permeable calcium dye; Texas Red, dye labels blood vessels in the brain for visualization*.

Vasomotor responses of the left and right common carotid arteries were examined next. Carotid arteries isolated from PKA KI mice exhibited impaired H_2_O_2_-induced vasodilation following pre-constriction with U-46619 compared with the WT preparations (Fig. 2A). In addition, acetylcholine-induced vasorelaxation was markedly attenuated in carotid arteries from PKA KI mice relative to WTs (Fig. 2B), consistent with previous work demonstrating an H_2_O_2_-dependent component in acetylcholine-mediated dilation [33], and further supporting the role of disulfide-PKARIα in modulating vascular tone of resistance arteries. There was no difference in the concentration-dependent relaxation of carotid arteries in response to the nitric oxide donor SNP (Fig. 2C), indicating preserved NO-cGMP-mediated vasodilation in the absence of PKARIα redox signaling. Responses to non-selective adenosine receptor stimulation, which should elevate cAMP levels, were also tested; however, in this case, significant relaxation after adenosine administration (not shown) or its more stable analogue, NECA (Suppl. Fig. 1C), could not be achieved in either genotype. There were no differences in the internal circumference of the left or right common carotid arteries at 100 mmHg, indicating no differences in blood vessel size (Suppl. Fig. 1D).

To test whether deficient oxidant-induced vasodilation in carotid arteries of the PKA KI mice was translated into relevant physiological *in vivo* changes, basal carotid blood flow parameters were assessed using Doppler ultrasound in anesthetized animals. Notably, age-matched PKA KI mice demonstrated lower velocity time integral and mean gradient in the left or right common carotid artery, with a trend toward lower mean velocity in the corresponding vessels of the PKA KI animals compared with the WT littermates (Fig. 2D, Suppl. Fig. 1E). All experiments to this point were conducted in young male mice (12-16 weeks). To broaden the age range and enhance translational relevance, additional experiments were performed in mid-aged mice (12-16 months), in parallel with young mice groups. Comparable to what was observed in young mice, the velocity-time integral, mean gradient, and mean velocity measured in the left common carotid artery were reduced in mid-aged PKA KI mice compared with age-matched WT controls, with a similar trend in the right common carotid artery (Fig. 2D, Suppl. Fig. 1E). Cardiac index (Fig. 2E) and other cardiac functional parameters (Suppl. Fig. 1F**)** were comparable between the genotypes, suggesting that the observed differences in flow are less likely to result from altered basal cardiac function and are instead due to changes in vascular tone caused by the loss of redox sensing and signaling through disulfide-PKARIα.

Systemic hypoxia triggers mitochondrial reactive oxygen species (ROS) production in brain cells [57], which may promote the oxidation of nearby proteins, particularly in the cerebral vasculature. To determine whether the PKARIα redox switching contributes to hypoxia-induced cerebral vasodilation *in vivo*, responses of penetrating cerebral arterioles to acute hypoxia were compared between genotypes using two-photon imaging in anesthetized, spontaneously breathing mice. Robust dilations of cerebral arterioles were observed in response to reductions in inspired oxygen (10% O_2_) in both PKA KI and wild-type mice; however, the vasodilatory responses did not differ between genotypes (Fig. 2F), consistent with little involvement of PKARIα-disulfide in hypoxic cerebrovascular vasodilation under experimental conditions of this study.

### 3.3 Cys17Ser PKARIα arteries demonstrate potentiated pressor responses to angiotensin II

**Fig. 3:**
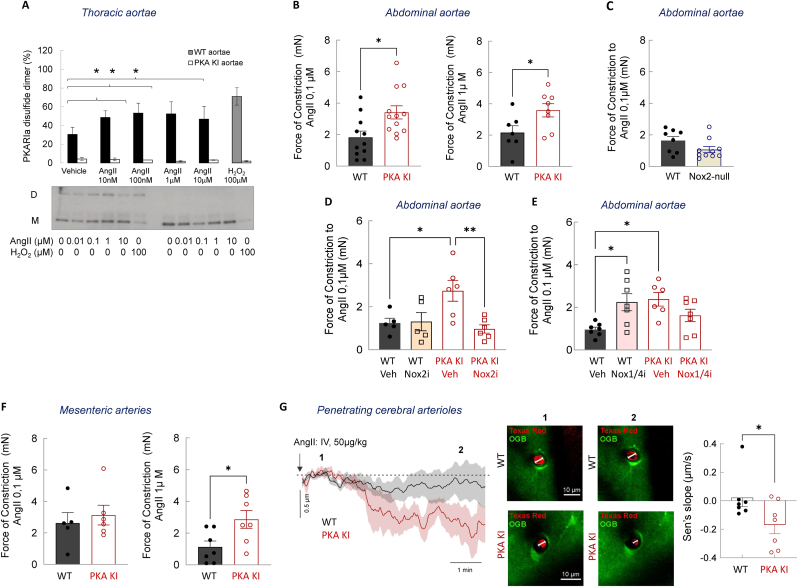
Comparison of *ex vivo* and *in vivo* vascular responses to angiotensin II acute treatment in wild-type or ‘redox-dead’ Cys17Ser PKARIα knock-in mice. **A.** Disulfide-PKARIα protein expression in thoracic aortae rings of WT or PKA KI mice subjected either to vehicle, AngII or H_2_O_2_ for 5 min (n = 3). **B–C.** Constriction of abdominal aortae rings from WT or PKARIα KI mice (**B**), or WT or Nox2-null mice (**C**) in response to a single bolus dose of AngII (n ≥ 7). **D-E.** Acute constriction of abdominal aortae rings from WT or PKA KI mice in response to a single bolus dose of AngII after a vehicle or Nox2-inhibitor GSK2795039 (25 μM, 30 min pre-incubation) pre-treatment (**D**), or a vehicle or Nox1/4-inhibitor GKT137831 (1 μM, 30 min pre-incubation) pre-treatment (**E**) (n ≥ 5). **F.** Constriction of mesenteric arteries from WT or PKA KI mice in response to a single bolus dose of AngII (n ≥ 5). **G.** Constriction of penetrating cerebral arterioles in anesthetized WT or PKA KI mice in response to a single bolus administration of AngII (50 μg/kg, i.v.) (n ≥ 7). *∗P< 0.05, ∗∗P< 0.01 vs. vehicle or respective WT. M, PKARIα monomer; D, PKARIα dimer; H*_*2*_*O*_*2*_*, hydrogen peroxide, acts a vasodilator; AngII, angiotensin II, acts as vasoconstrictor; WT, wild type; PKA KI, Cys17Ser PKARIα knock-in mice; Nox2-null, Nox2 knockout mice; OGB, Oregon Green 488 BAPTA-1, cell permeable calcium dye; Texas Red, dye labels blood vessels in the brain for visualization.*

AngII is a potent vasoconstrictor and a major contributor to systemic hypertension [58]. In addition to its contractile effects, AngII activates oxidant signaling pathways that may partially counterbalance vasoconstriction by generating H_2_O_2_, which in turn can engage vasodilatory mechanisms, including those mediated by oxidative activation of PKARIα. To determine whether AngII promotes oxidation in the vasculature, we evaluated PKARIα redox status after treatment with this peptide hormone. PKARIα disulfide formation was evident in rat aortic smooth muscle cells treated with AngII (Suppl. Fig. 2A), and in aortae isolated from WT, but not PKA KI, mice (Fig. 3A), in agreement with AngII-driven activation of NADPH oxidase (Nox) enzymes and H_2_O_2_ production. Isolated arteries do not sustain the prolonged contractile response elicited by a dose-response to AngII *in vivo*, owing in part to internalization and redistribution of AT_1_ receptors, a process thought to limit vascular injury during persistent peptide exposure [59]. As a result, conventional concentration-response curves are challenging to obtain in organ bath preparations. Therefore, vasomotor responses to a single bolus dose of AngII were assessed. It was found that under these conditions, abdominal aorta rings from PKA KI mice exhibited greater constriction in response to 0.1 or 1 μM AngII compared with the WT littermate controls (Fig. 3B, Suppl. Fig. 2B), supporting a loss of an oxidant-dependent vasodilatory component in the absence of PKARIα redox signaling.

To identify the enzymatic source of the oxidants responsible for this counter-regulatory effect, Nox isoforms were examined as candidate contributors. Given their established roles in vascular redox signaling, Nox2-and Nox4-derived oxidants were initially evaluated [60]. However, AngII-induced constriction of abdominal aortae was comparable between Nox2-null and WT mice (Fig. 3C) and unaffected by acute pharmacological inhibition of Nox2 with GSK2795039 (Fig. 3D), indicating that Nox2-derived oxidants are not required for the vasodilatory component accompanying acute AngII-induced contraction in this vascular bed. To extend these findings and enhance translational relevance, AngII responses were also assessed in mid-aged mice. Consistent with observations in young animals (Fig. 3B), abdominal aortae from mid-aged PKA KI mice exhibited enhanced constriction to 0.1 μM AngII compared with age-matched WT controls (Suppl. Fig. 2C). In contrast, AngII-induced responses were similar between Nox2-null and WT mid-aged mice (Suppl. Fig. 2D), and between Nox4-null and WT mid-aged mice (Suppl. Fig. 2E), further excluding an essential role for either Nox2 or Nox4 in mediating the oxidant-dependent bolus effects of AngII that partially offset vasoconstriction.

Strikingly, pharmacological inhibition of Nox1/4 with the dual selective inhibitor GKT137831 potentiated AngII-induced constriction in abdominal aortae from WT mice but had minimal effect in vessels from KI littermates, which already exhibited exaggerated constriction (Fig. 3E). The absence of an additional effect in PKA KI vessels indicates that the Nox1/4-sensitive oxidant signal converges on PKARIα. Because PKARIα oxidation occurs in WT but not PKA KI vessels and AngII-induced vasoconstriction is similar in the abdominal aorta of Nox-4 null and WT mice, these data strongly implicate Nox1-derived oxidants as upstream mediators of AngII-induced PKARIα disulfide formation, thereby engaging a counter-regulatory vasodilatory pathway. Consistent with this interpretation, mesenteric arteries from PKA KI mice displayed augmented constriction to 1 μM AngII compared with WT controls (Fig. 3F). Furthermore, it was also found that penetrating cerebral arterioles from PKA KI mice constricted in response to an acute intravenous AngII (50 μg/kg), whereas WT mice showed no cerebrovascular response to AngII (Fig. 3G).

Collectively, these findings establish that PKARIα oxidation within the vasculature functions as a Nox-dependent counter-regulatory mechanism that limits AngII-induced vasoconstriction across multiple vascular beds. Both conduit and resistance arteries and resistance arterioles from PKA KI mice exhibit impaired H_2_O_2_-dependent vasodilation and exaggerated AngII-induced constriction compared with WT controls, underscoring the PKARIα disulfide-dimer oxidation as a critical brake on acute AngII-driven vasoconstriction. We therefore next examined whether this mechanism also modulates sustained vasopressor responses and blood pressure elevation *in vivo*.

### 3.4 Cys17Ser PKARIα KI mice are adapted to be normotensive

**Fig. 4:**
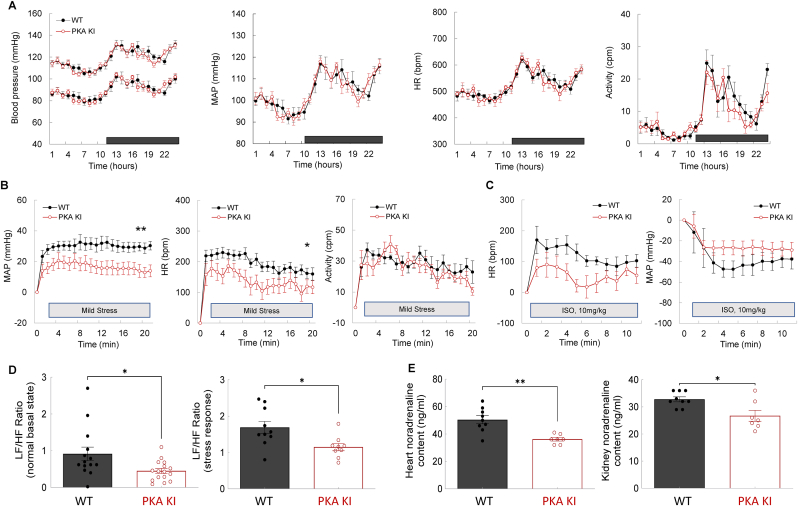
Basal blood pressure and stress-responses in wild-type or ‘redox-dead’ Cys17Ser PKARIα knock-in mice. **A.** Basal systolic, diastolic, mean blood pressure, heart rate and locomotor activity in conscious freely moving WT or PKA KI mice over 24 h. The horizontal black bar (12–24 h) indicates the nocturnal active phase for mice (n ≥ 14). **B.** Heart rate, mean blood pressure and activity during the period of mild stress, when conscious freely moving WT or PKA KI mice were subjected to a new environment for 20 min (n = 9). **C.** Heart rate and mean blood pressure responses in conscious WT or PKA KI mice, acutely (e.g., for 10 min) treated with adrenergic agonist isoprenaline (intraperitoneal injection at a dose 10 mg/kg) (n = 7). **D.** Low-to-high frequency ratio assessed basally (n ≥ 14) and after a mild stress (n = 9) in conscious WT or PKA KI mice. **E.** Basal norepinephrine level in heart (left) or kidney (right) tissues of WT or PKA KI mice (n ≥ 7). *∗P< 0.05, ∗∗P< 0.01 vs. vehicle or respective WT. WT, wild type; PKA KI, Cys17Ser PKARIα knock-in; SBP, systolic blood pressure; DBP, diastolic blood pressure; MAP, mean arterial pressure; ISO, adrenergic agonist isoprenaline; HR, heart rate; LF/HF ratio, low-to-high frequency ratio, as a frequency domain parameter of heart rate variability.*

Contrary to our expectation, systolic and diastolic blood pressure, mean arterial pressure, heart rate and locomotor activity were comparable between PKA KI and WT mice under basal conditions (Fig. 4A). Although this finding appears unexpected in light of the impaired oxidant-dependent vasodilation observed *ex vivo*, systemic arterial pressure and tissue perfusion are determined by the integrated balance between cardiac output and systemic vascular resistance. These parameters are, in turn, regulated by multiple overlapping control systems including the baroreceptor reflex, autonomic nervous system activity, the renin-angiotensin system, nitric oxide signaling and intrinsic myogenic responses [61]. Compensation within one or more of these pathways may therefore maintain basal blood pressure homeostasis despite the absence of PKARIα redox sensing and signaling. To explore this possibility, basal stroke volume, cardiac output and fractional shortening were assessed and found to be comparable between genotypes (Fig. 2E, Suppl. Fig. 1F). These data indicate that the normotensive phenotype of the transgenic mice, anticipated to be hypertensive based on their impaired arterial vasodilation, is not attributable to an adaptive reduction in cardiac output.

To further explore potential adaptive mechanisms underlying the normotensive phenotype of PKA KI mice, neuronal autonomic responses were examined and compared with those of WT littermates. ‘Redox-dead’ mice expressing Cys17Ser PKARIα displayed attenuated increases in both heart rate and blood pressure in response to acute mild stress, induced by exposure to a novel environment (Fig. 4B). In addition, transgenics showed a trend toward a reduced chronotropic response following bolus administration of the β-adrenergic agonist isoproterenol compared with WT controls (Fig. 4C). Taken together, these findings imply altered autonomic cardiovascular regulation in the transgenic mice. One possible explanation for these observations is that PKA KI mice developed increased baroreceptor sensitivity, and the blood pressure increase would have been larger if their heart rate response during stress matched that of the WTs. Sympathetic neurons innervate both the heart and the vasculature and play a central role in regulating cardiac output and vascular tone [62]. Postganglionic sympathetic fibers, which localize to the adventitial-medial border of most arteries, release norepinephrine to promote vasoconstriction *via* activation of α_1_-and α_2_-adrenoceptors on vascular smooth muscle cells [63]. Accordingly, increased sympathetic outflow enhances vasoconstriction, whereas withdrawal of sympathetic tone favors vasodilation. To further assess autonomic balance [64], HRV analysis was performed using high-resolution remote radiotelemetry recordings. Frequency-domain HRV analysis revealed a marked autonomic alteration in PKA KI mice, characterized by an approximately 50% reduction in sympathetic dominance, as indexed by the low-to-high frequency (LF/HF) ratio, evident both under basal conditions and following exposure to acute stress (Fig. 4D). These findings indicate a sustained attenuation of sympathetic drive in the transgenic mice, which would be expected to limit excessive increases in heart rate and blood pressure during adrenergic stimulation. Consistent with reduced sympathetic dominance at the tissue level, norepinephrine content was significantly lower in the hearts and kidneys of PKA KI mice compared with WT controls (Fig. 4E). In contrast, circulating plasma norepinephrine concentrations were comparable between genotypes (Suppl. Fig. 3A), possibly indicating that overall systemic circulating adrenergic tone is preserved. Such tissue-level *in vivo* autonomic adaptation may, at least in part, explain the lack of hypertension anticipated in the PKA KI mice, despite their arteries exhibiting exacerbated constrictor responses to vasopressors *ex vivo*.

Although basal arterial pressure was similar between WT and PKA KI mice, it remained possible that differences in blood pressure regulation might emerge under sustained cardiovascular stress, when compensatory mechanisms could become limiting. Consequently, the effects of chronic AngII infusion on blood pressure were examined next.

### 3.5 Cys17Ser PKARIα KI mice develop exacerbated hypertension and pressor-induced hypertrophy in response to chronic infusion of angiotensin II

**Fig. 5:**
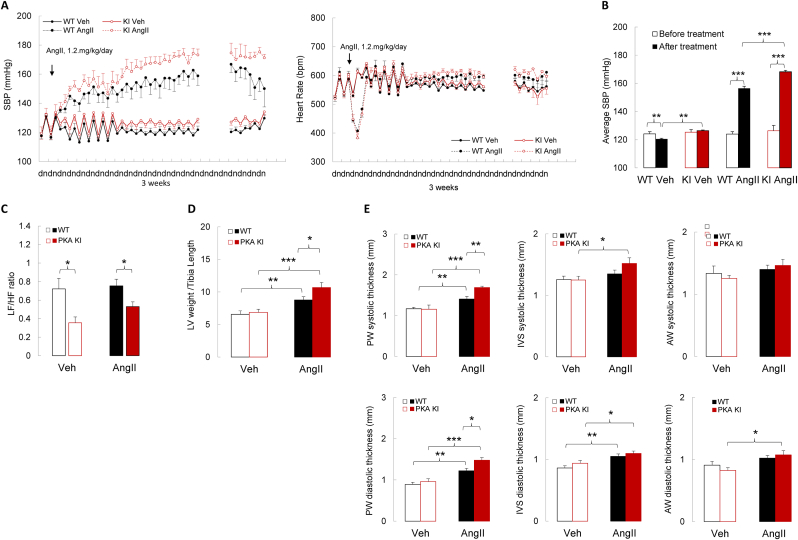
Hemodynamic responses of wild-type or ‘redox-dead’ Cys17Ser PKARIα knock-in mice to chronic infusion of angiotensin II. **A.** Systolic blood pressure and heart rate responses in conscious WT or PKA KI mice treated with vehicle or AngII (1.2 mg/kg/day) over a period of three weeks (n ≥ 7). **B–C.** Averaged systolic blood pressure (**B**) and low-to-high frequency ratio, as a frequency domain parameter of heart rate variability (**C**) in WT or PKA KI mice before and after AngII treatment (n ≥ 7). **D-E.** Left ventricular weight/tibial length ratio (**D**) and posterior wall, intraventricular septum, and anterior wall systolic and diastolic thicknesses assessed by echocardiography (**E**) in anesthetized WT or PKA KI mice, after three weeks of treatment either with vehicle or angiotensin II (n ≥ 7). *∗P< 0.05, ∗∗P< 0.01, ∗∗∗P< 0.001 vs. vehicle or respective WT. WT, wild type; PKA KI, Cys17Ser PKARIα knock-in; SBP, systolic blood pressure; AngII, angiotensin II, acts as vasoconstrictor; LF/HF ratio, low-to-high frequency ratio, as a frequency domain parameter of heart rate variability; PW, posterior wall; IVS, intraventricular thickness; AW, anterior wall.*

WT and PKA KI mice were administered a pressor dose of AngII (1.2 mg/kg/day) for three weeks to assess cardiovascular responses under sustained hypertensive stress. As expected, both genotypes developed elevated arterial pressure within 2-3 days of AngII exposure, and hypertension was maintained throughout the duration of treatment (Fig. 5A–B). Notably, however, the increase in systolic blood pressure was significantly greater in PKA KI mice compared to WT controls. Importantly, heart rate responses were comparable between genotypes (Fig. 5A–B), meaning that exaggerated pressor responses to AngII cannot be attributed to differences in chronotropic regulation. Furthermore, analysis of HRV revealed that AngII treatment did not increase sympathetic dominance in either genotype (Fig. 5C). This is in line with previous work demonstrating that AngII-induced hypertension is not driven by increased sympathetic nerve activity to key vascular beds in rodents [65], and with human studies showing no increase in sympathetic activity, assessed by norepinephrine spillover, during AngII infusion [66]. Importantly, the reduced sympathetic dominance observed basally in PKA KI mice, indexed by a lower LF/HF ratio, was preserved during chronic AngII infusion (Fig. 5C). Altogether, these findings indicate that the hypertensive response to AngII in the absence of disulfide-PKARIα signaling arises primarily from its direct vascular actions rather than from augmented sympathetic outflow.

To evaluate the cardiac consequences of sustained AngII-induced hypertension, echocardiographic assessments were performed before and after treatment. As expected, cardiac mass due to pressure-induced left ventricular hypertrophy was more pronounced in the PKA KI mice (Fig. 5D), as they developed a greater and sustained hypertensive response during AngII exposure. This was accompanied by a greater increase in posterior wall thickness, measured in both systole and diastole, compared with WT controls following AngII treatment (Fig. 5E). In contrast, intraventricular septal thickness in diastole increased similarly in both genotypes, whereas systolic septal thickening was observed only in PKA KI mice (Fig. 5E). Anterior wall thickness in diastole was increased only in AngII-treated PKA KI mice, while no changes in systolic anterior wall thickness were detected in either genotype (Fig. 5E).

The reductions in ejection fraction, stroke volume, cardiac output or fractional shortening after AngII administration were similar between the genotypes (Suppl. Fig. 3B–C), reflecting similar degrees of functional cardiac adaptation to chronic pressure overload. The renal function marker, blood urea nitrogen, increased to a similar extent in WT and PKA KI mice following chronic pressor treatment, while hematocrit and hemoglobin levels were significantly elevated in AngII-treated PKA KI mice compared with vehicle-treated animals, with a similar but non-significant trend observed in WT mice (Suppl. Fig. 3D). Thus, a loss of disulfide-PKARIα signaling exacerbates the hypertensive and cardiac remodeling responses to chronic AngII exposure, despite preserved autonomic adaptation. This is once again consistent with the absence of an oxidant-dependent vasodilatory mechanism that would normally offset hormone-induced vasoconstriction and limit pressure overload-induced cardiovascular remodeling.

## 4 Discussion

Redox regulation of vascular function through H_2_O_2_ and/or other redox-active molecules has been a subject of significant scientific interest [67], with several effector proteins [10,34,60,67], including kinases [10], being identified as downstream redox sensors mediating physiological responses. PKARIα plays an essential role in vascular physiology and blood pressure regulation *via* its canonical cAMP-dependent kinase function. Here, we demonstrate a novel non-canonical role for PKARIα: formation of interprotein disulfide dimers between Cys17 and Cys38 functions as a redox sensor that drives oxidant-induced vasodilation and lowers blood pressure.

The roles of NO and prostacyclin-triggered vasodilatory pathways in regulating blood pressure are well-defined [36,[68], [69], [70]]. For example, it is established that PKGI mediates NO-dependent and NO-independent mechanisms [33,50,69,[71], [72], [73]]. Previously, we demonstrated that oxidants modulate endothelium-derived hyperpolarizing factor-dependent vasodilation, in part, by promoting an interprotein disulfide bond between the homodimeric subunits of PKGIα [33,44,73], thereby decreasing the Ca^2+^ concentration in vascular smooth muscle cells [74]. These mechanistic studies were complemented by the generation of ‘redox-dead’ Cys42Ser PKGIα knock-in mice that were unable to sense or transduce oxidant signals by inducing interprotein disulfide formation, as they were engineered to have the essential thiol (SH) side chain replaced by a hydroxyl (OH) moiety, and as a result these mice exhibit deficient oxidant-mediated arterial vasodilation [33,35,44,45]. Notably, though, significant oxidant-induced dilation was still evident in arteries from Cys42Ser PKGIα knock-in mice [33,44], consistent with additional oxidant-signaling pathways contributing to the vasodilation.

In this regard, PKARIα was a rational target to assess, as PKA activation couples to vasorelaxation [36,37] and its oxidation to the interprotein disulfide dimer state is associated with substrate targeting and phosphorylation [25,29]. We previously demonstrated that PKARIα oxidation contributes to nitrosocysteine-induced vasodilation, accompanied by RIα disulfide dimer formation [56]. This finding is consistent with the established role of PKARIα in mediating vasodilatory responses to endogenous agonists such as epinephrine and prostacyclin, which engage Gs-coupled receptors to increase cAMP and activate PKA, ultimately reducing systemic and pulmonary arterial pressure [36,37,75]. Using ‘redox-dead’ Cys17Ser RIα mice, unable to sense or transduce oxidant signals through PKARIα-disulfide dimer, as an experimental tool, we demonstrate here that both conduit and resistance arteries employ PKARIα oxidation to promote vasodilation. We show that loss of PKARIα redox sensing and signaling selectively impaired H_2_O_2_- and acetylcholine-dependent vasodilation while preserving NO-dependent relaxation. Our results strongly support the causal role of the PKARIα redox sensor as a physiological vascular mechanism that promotes arterial relaxation and counterbalances vasoconstriction, particularly under oxidative stress, as seen in chronic hypertension.

Intracellular H_2_O_2_ is thought to be maintained at nanomolar steady-state levels under physiological conditions [76,77], although, in this study, to simulate intracellular ROS production, aortic smooth muscle cells or isolated arteries were treated with H_2_O_2_ at nanomolar and low to high micromolar concentrations. These concentrations used to elicit measurable redox responses, including PKARIα disulfide dimer formation and vasodilation, were chosen based on our prior work and that of others [33,35,39,44,[76], [77], [78], [79]] based on the rapid (seconds to minutes) ROS scavenging by tissue or cell culture media antioxidant systems and to account for a possible rapid degradation of H_2_O_2_ in the extracellular space.

Arterial tone is essential for controlling blood flow and tissue perfusion. While the aorta transports oxygenated blood from the heart to the organs and tissues, carotid arteries, which branch from the aortic arch, act as small conduits to deliver oxygenated blood to the brain and also contribute to resistance that can influence cerebral perfusion. Because the brain is susceptible to perturbations in oxygen delivery, carotid vascular dysfunction is directly relevant to pathological conditions such as hypoxic injury and ischemic stroke. Notably, deficient oxidant-induced vasodilation in the common carotid arteries of PKA KI mice was associated with lower blood flow velocity parameters in these vessels. There was a trend toward lower carotid blood flow in mid-aged WT mice, consistent with the reported age-related decline in carotid and cerebral blood flow in humans [80]. Interestingly, these parameters remained similar between young and mid-aged transgenic mice and, on average, were lower than those observed in young wild-type controls, potentially indicating features of early vascular aging in PKA KI mice. No differences in cardiac performance or vessel diameter were detected between genotypes, reinforcing the fact that the reduced carotid blood flow in KI mice reflects altered vasomotor properties rather than changes in cardiac output or vascular structure. Taken together, these data demonstrate the physiological relevance of PKARIα oxidative signaling in regulating carotid blood flow and cerebral perfusion.

Based on our previous finding that *in vivo* hypoxia induces disulfide-PKGIα in lung tissue through Nox4-or extracellular superoxide dismutase-dependent mechanisms, we investigated whether the PKARIα redox switching mediates hypoxia-induced cerebral vasodilation [51]. Hypoxia can potentiate H_2_O_2_ generation by xanthine oxidase in vascular endothelial cells [81,82], a process partially mediated by adenosine-dependent signaling [81]. Mitochondrial H_2_O_2_ production in the brain has been shown to increase transiently during hypoxia [57]. Astrocytes have been proposed to function as oxygen sensors, as hypoxia inhibits astroglial mitochondrial respiration, leading to mitochondrial depolarization and ROS production [83]. In principle, such ROS could diffuse from astrocytic endfeet to adjacent vascular cells and contribute to vasodilation. In contrast, our previous work also demonstrated a reduction of sulfenic acid to the thiol state in the heart under hypoxic conditions, likely reflecting decreased oxidant production [84]. A more reduced thiol state of cyclin-dependent kinase 4 was observed in lungs from patients with chronic pulmonary vascular disease [85], which is often associated with hypoxia and metabolic reprogramming. Indeed, metabolic adaptation to hypoxia is usually associated with a pro-reducing environment rather than a pro-oxidizing one, along with other related metabolic changes [86]. The exact redox response to hypoxia is likely more complex and depends on oxygen concentration, duration, cell type and the model system.

Most mechanistic studies of acute hypoxic cerebral vasodilation emphasize endothelium-derived factors, including NO, prostacyclin, and metabolic vasodilators such as adenosine, potassium, and hydrogen ions [87], as the primary mediators of the vasodilatory response, rather than ROS. In our study, reproducible dilations of penetrating cerebral arterioles occurred immediately after the onset of the hypoxic stimulus; however, responses were comparable between wild-type and PKA KI mice. The wild-type group exhibited unusually high variability in responses, reflecting possible limitations of the model system employed. These findings are consistent with adenosine- and NO-mediated signaling as the primary regulators of acute hypoxic cerebral vasodilation [46,88], with little contribution from H_2_O_2_-mediated pathways or PKARIα oxidation in this context, and point to a role for this redox-regulated kinase in maintaining basal vascular tone rather than mediating acute hypoxic vasodilation. Nevertheless, the small size and delicate structure of cerebral vessels precluded assessment beyond hypoxic or acute AngII responses with standard myography techniques.

Remarkably, despite a robust PKARIα-disulfide-dependent deficit in vasodilation across multiple vascular beds, transgenic mice preserve normotension both at rest and under mild environmental stress. Blood pressure recordings *via* gold-standard remote radiotelemetry confirmed values consistent with healthy adult mice, as we [33,44,45] and others [89] reported. Although surprising, this likely underscores the essential role of blood pressure control in maintaining homeostasis and protecting organs. Consequently, intrinsic, neural, and hormonal mechanisms can modulate the hemodynamic state to facilitate adaptation to both internal and environmental challenges [89]. Indeed, such adaptation was previously observed in mice heterozygous for the alpha subunit of the epithelial sodium channel, in which activation of the renin-angiotensin-aldosterone system compensated for the loss of this protein in the distal tubules of the kidney, thereby maintaining normal blood pressure [90]. Phospholemman knock-in mice, in which this small regulatory protein cannot be phosphorylated at serines 63, 68, and 69, exhibit chronic autonomic adaptation of baroreceptor sensitivity, allowing them to retain normotension and low pressor responses to exogenous phenylephrine or environmental stress *in vivo*, or during age-dependent hypertension, despite enhanced vasoconstriction of the peripheral vasculature assessed *ex vivo*. [50]. In our study, autonomic adaptation in PKA KI mice is evidenced in the significantly reduced LF/HF ratio (at rest or under mild stress), consistent with a chronic adaptive reduced sympathetic dominance and altered autonomic balance, limited chronotropic response to environmental stress and beta-adrenergic stimulation, along with decreased regional heart and kidney norepinephrine noradrenaline spillover, compared with wild-type controls. Together, these results support the presence of a tissue-level autonomic adaptation in PKA KI mice that allow them to maintain normotension despite the absence of PKARIα-dependent modulation of vasodilation. This autonomic adaptation, in which sympathetic dominance is attenuated, is therefore likely to balance the lack of vasodilation and excessive vasoconstriction observed in PKA KI mice to sustain *in vivo* organ perfusion. Future work may investigate this further by direct assessment of renal sympathetic nerve activity and/or by measuring baroreceptor and chemoreceptor control of cardiovascular parameters by specifically blocking parasympathetic and sympathetic components (e.g., by assessing intrinsic heart rate). Alternatively, a vascular-specific PKARIα transgenic could be generated to evaluate blood pressure and autonomic function as performed here.

Angiotensin II is a vasopressor that acts on vascular AT1 receptors to elicit vasoconstriction [58]. Here we demonstrate that AngII-induced constriction is augmented in vessels from PKA KI mice compared with WT littermates and present evidence that, in addition to its contractile signaling through phospholipase C, AngII activates Nox protein-dependent oxidant signaling pathways that partially offset vasoconstriction by generating H_2_O_2_ that oxidizes PKARIα. Nox1, Nox2 and Nox4 have been implicated as sources of ROS that modulate vascular responses to AngII [[91], [92], [93]], including our work showing Nox4-dependent PKARIα oxidation in response to pro-angiogenic growth factor stimulation [39]. In this study, we observe a potentiation of AngII-constriction when both Nox1 and Nox4 activity are inhibited, but only in the WT mice, not in PKA KIs. In light of the Nox4-null aorta having a similar AngII-constriction to wild-type controls, it is plausible that, at least in the bolus treatment, in addition to AT1-receptor mediated acute vasoconstriction, AngII also stimulates Nox1 that generates H_2_O_2_ [91,93], which is well known to dilate resistance arteries [32].

AngII is a key determinant of blood pressure homeostasis, and dysregulation of its signaling can cause pathogenic hypertension [92]. Consistent with this, exposure to AngII elicited more pronounced systemic hypertension and more severe pressure-overload cardiac hypertrophy in the PKA KI mice. Notably, vehicle-treated PKA KI mice also showed a modest trend toward higher basal blood pressure than WT mice, which became apparent about one week after the start of vehicle infusion. This may reflect subtle impairment of autonomic compensatory mechanisms that are still stabilizing during the early post-pump implantation period and become more evident in the transgenics under additional physiological or neurohormonal stress. Together, these observations indicate that oxidant-dependent activation of PKARIα functions as an intrinsic vascular counter-regulatory mechanism, promoting arterial relaxation and limiting excessive vasoconstriction and hypertrophy during conditions of elevated oxidant signaling, such as those encountered in chronic hypertension. Although there is evidence for AngII causing systemic hypertension partially due to increased production of oxidants [94], our data point to oxidants produced during hormone administration causing vasodilation instead of constriction. Indeed, the exacerbated AngII-induced hypertension in the PKA KI is consistent with observations in isolated arteries and, overall, supports PKARIα oxidation as an important mechanism of vasodilation that operates *in vivo* to regulate blood pressure. Canonical PKA activation mediates an adaptive mechanism by phosphorylating eNOS at Ser633 and Ser1177 in response to activation of AT_2_ receptors by AngII [95], suggesting that PKA may increase NO to offset hypertension, although the extent to which this mechanism may be employed by disulfide-PKARIα is unknown. Our findings extend the emerging evidence that redox switching, *via* oxidation of PKARIα to its disulfide-dimerized form, regulates the cardiovascular system [29,31,39,56].

In summary, we identify a redox sensor in the regulatory RIα subunit of type I PKA that forms an interprotein disulfide dimer coupling oxidant signaling to arterial dilation and blood pressure lowering, a previously unrecognized mechanism of oxidant-dependent vasoregulation. This oxidation event acts as a pressure-limiting mechanism during AngII-induced hypertension by restraining vasoconstriction and attenuating cardiac hypertrophy. These findings highlight PKARIα as a potential target for thiol-based therapies to treat hypertension.

## CRediT authorship contribution statement

**Olena Rudyk:** Conceptualization, Data curation, Formal analysis, Funding acquisition, Investigation, Methodology, Project administration, Resources, Software, Supervision, Validation, Visualization, Writing – original draft, Writing – review & editing. **Alice Braga:** Formal analysis, Investigation, Writing – review & editing. **Naomi Wheatcroft:** Formal analysis, Investigation, Methodology. **Mattia Bonzanni:** Formal analysis, Methodology, Software. **Oleksandra Prysyazhna:** Formal analysis, Investigation. **Michelle N.A. Korneh:** Investigation. **Hannah L.H. Green:** Investigation. **Yang Zhou:** Investigation. **Min Zhang:** Investigation, Resources, Writing – review & editing. **Alexander V. Gourine:** Formal analysis, Investigation, Methodology, Resources, Writing – review & editing. **Philip Eaton:** Conceptualization, Funding acquisition, Resources, Writing – review & editing.

## Declaration of competing interest

The authors declare that they have no known competing interests that could have appeared to influence the work reported in this paper.

## Appendix A Supplementary data

Supplementary Figure1. *In vitro* cell responses, *ex vivo* and *in vivo vascular responses* of wild-type or ‘redox-dead’ Cys17Ser PKARIα knock-in mice. A. Disulfide-PKARIα protein expression in thoracic aortae rings of WT mice subjected either to a vehicle or 50, 100, 200 and 500 μM H_2_O_2_ treatment for 30 min. **B.** Dose-dependent constriction of aortic rings from WT or PKA KI mice in response to U-46619. **C.** NECA-induced dose-dependent response of carotid arteries (pre-constricted with U-46619), from WT or PKA KI mice **D.** Internal circumference of left and right carotid common carotid arteries assessed by myography in WT and PKA KI mice. **E.** Basal carotid arterial flow parameters assessed by ultrasound Doppler in anesthetized WT or PKA KI mice. **F.** Basal cardiac function parameters assessed by ultrasound in anesthetized WT or PKA KI mice. *∗P< 0.05, ∗∗∗P< 0.001 versus vehicle or a respective WT. SMCs, smooth muscle cells; WT, wild type; PKA KI, Cys17Ser PKARIα knock-in mice; H*_*2*_*O*_*2*_*, hydrogen peroxide, acts a vasodilator; U-46619, stable synthetic analogue of the prostaglandin PGH2, acts as a thromboxane receptor agonist; NECA, 5′-N-Ethylcarboxamidoadenosine, non-selective adenosine receptor agonist.* Supplementary Figure2. *In vitro* and *ex vivo* vascular responses to angiotensin II in wild-type or ‘redox-dead’ Cys17Ser PKARIα knock-in mice. **A.** Disulfide-PKARIα protein expression in aortic smooth muscle cells subjected to vehicle or AngII for 15 min (left). Disulfide-PKARIα protein expression in aortae rings of WT mice subjected either to vehicle, AngII or H_2_O_2_ for 5 min (right). **B.** Representative traces obtained by myography from WT or PKA KI mice in abdominal aortae or mesenteric arteries. Acute constriction of abdominal aortae rings in response to a single bolus dose of AngII in mid-aged WT or PKA KI mice **(C)**, mid-aged Nox2-null mice **(D)** or mid-aged Nox4-null mice **(E)**. *∗P< 0.05 versus vehicle or respective WT. M, PKARIα monomer; D, PKARIα dimer; H*_*2*_*O*_*2*_*, hydrogen peroxide; AngII, angiotensin II; WT, wild type; PKA KI, Cys17Ser PKARIα knock-in mice; Nox2-null, Nox2 knockout mice; Nox4-null, Nox4 knockout mice*. Supplementary Figure3. Hemodynamic responses of wild-type or ‘redox-dead’ Cys17Ser PKARIα knock-in mice to chronic infusion of angiotensin II. **A.** Basal norepinephrine level in blood plasma of WT or PKA KI mice. **B.** Ejection fraction, stroke volume, fractional shortening and cardiac output assessed by echocardiography in anesthetized WT or PKA RIα KI mice, after three weeks of treatment either with vehicle or AngII. **C.** Representative M-mode cardiac function images obtained by echocardiography in anesthetized WT (left panel) or PKA RIα KI (right panel) mice after 3 weeks of vehicle (top two panels) or AngII (1.2 mg/kg/day, bottom two panels) chronic treatment. **D.** Blood urea nitrogen, hematocrit and hemoglobin level in blood of WT or PKARIα KI mice, after three weeks of treatment either with vehicle or angiotensin-II. *n=6–8/group, ∗P< 0.05, ∗∗P< 0.01, ∗∗∗P< 0.001 versus vehicle or respective WT. WT, wild type; PKA KI, Cys17Ser PKARIα knock-in; AngII, angiotensin II.*Multimedia component 1

## References

[1] Johansson B.B. (1999). Hypertension mechanisms causing stroke. Clin. Exp. Pharmacol. Physiol..

[2] Kokubo Y., Matsumoto C. (2017). Hypertension is a risk factor for several types of heart disease: review of prospective studies. Adv. Exp. Med. Biol..

[3] Ciruzzi M., Pramparo P., Rozlosnik J., Zylberstjn H., Delmonte H., Haquim M., Abecasis B., de La Cruz Ojeda J., Mele E., La Vecchia C., Schargrodsky H. (2001). Hypertension and the risk of acute myocardial infarction in Argentina. The Argentine Factores de Riesgo Coronario en America del Sur (FRICAS) Investigators. Prev. Cardiol..

[4] Emdin C.A., Anderson S.G., Callender T., Conrad N., Salimi-Khorshidi G., Mohseni H., Woodward M., Rahimi K. (2015). Usual blood pressure, peripheral arterial disease, and vascular risk: cohort study of 4.2 million adults. BMJ.

[5] Makin A., Lip G.Y., Silverman S., Beevers D.G. (2001). Peripheral vascular disease and hypertension: a forgotten association?. J. Hum. Hypertens..

[6] Whelton P.K., Klag M.J. (1989). Hypertension as a risk factor for renal disease. Review of clinical and epidemiological evidence. Hypertension.

[7] Kobeissi E., Hibino M., Pan H., Aune D. (2019). Blood pressure, hypertension and the risk of abdominal aortic aneurysms: a systematic review and meta-analysis of cohort studies. Eur. J. Epidemiol..

[8] de Boer I.H., Bangalore S., Benetos A., Davis A.M., Michos E.D., Muntner P., Rossing P., Zoungas S., Bakris G. (2017). Diabetes and hypertension: a position statement by the American diabetes association. Diabetes Care.

[9] Touyz R.M., Rios F.J., Alves-Lopes R., Neves K.B., Camargo L.L., Montezano A.C. (2020). Oxidative stress: a unifying paradigm in hypertension. Can. J. Cardiol..

[10] Cuello F., Eaton P. (2019). Cysteine-based redox sensing and its role in signaling by cyclic nucleotide-dependent kinases in the cardiovascular system. Annu. Rev. Physiol..

[11] Taylor S.S., Kim C., Vigil D., Haste N.M., Yang J., Wu J., Anand G.S. (2005). Dynamics of signaling by PKA. Biochim. Biophys. Acta.

[12] Bers D.M., Xiang Y.K., Zaccolo M. (2019). Whole-cell cAMP and PKA activity are epiphenomena, nanodomain signaling matters. Physiology (Bethesda).

[13] Bers D.M., Despa S. (2009). Na/K-ATPase--an integral player in the adrenergic fight-or-flight response. Trends Cardiovasc. Med..

[14] Murthy K.S. (2006). Signaling for contraction and relaxation in smooth muscle of the gut. Annu. Rev. Physiol..

[15] Kandel E.R. (2012). The molecular biology of memory: camp, PKA, CRE, CREB-1, CREB-2, and CPEB. Mol. Brain.

[16] Ravnskjaer K., Madiraju A., Montminy M. (2016). Role of the cAMP pathway in glucose and lipid metabolism. Handb. Exp. Pharmacol..

[17] Schmitt J.M., Stork P.J. (2001). Cyclic AMP-Mediated inhibition of cell growth requires the small G protein Rap1. Mol. Cell Biol..

[18] Wehbi V.L., Tasken K. (2016). Molecular mechanisms for cAMP-Mediated immunoregulation in T cells - role of anchored protein kinase A signaling units. Front. Immunol..

[19] Taylor S.S., Ilouz R., Zhang P., Kornev A.P. (2012). Assembly of allosteric macromolecular switches: lessons from PKA. Nat. Rev. Mol. Cell Biol..

[20] Diviani D., Reggi E., Arambasic M., Caso S., Maric D. (2016). Emerging roles of A-kinase anchoring proteins in cardiovascular pathophysiology. Biochim. Biophys. Acta.

[21] Caricati-Neto A., Garcia A.G., Bergantin L.B. (2015). Pharmacological implications of the Ca(2+)/cAMP signaling interaction: from risk for antihypertensive therapy to potential beneficial for neurological and psychiatric disorders. Pharmacol Res Perspect.

[22] Taylor S.S., Kim C., Cheng C.Y., Brown S.H., Wu J., Kannan N. (2008). Signaling through cAMP and cAMP-dependent protein kinase: diverse strategies for drug design. Biochim. Biophys. Acta.

[23] Smith F.D., Esseltine J.L., Nygren P.J., Veesler D., Byrne D.P., Vonderach M., Strashnov I., Eyers C.E., Eyers P.A., Langeberg L.K., Scott J.D. (2017). Local protein kinase A action proceeds through intact holoenzymes. Science.

[24] Kinderman F.S., Kim C., von Daake S., Ma Y., Pham B.Q., Spraggon G., Xuong N.H., Jennings P.A., Taylor S.S. (2006). A dynamic mechanism for AKAP binding to RII isoforms of cAMP-dependent protein kinase. Mol. Cell.

[25] Brennan J.P., Bardswell S.C., Burgoyne J.R., Fuller W., Schroder E., Wait R., Begum S., Kentish J.C., Eaton P. (2006). Oxidant-induced activation of type I protein kinase A is mediated by RI subunit interprotein disulfide bond formation. J. Biol. Chem..

[26] Brennan J.P., Wait R., Begum S., Bell J.R., Dunn M.J., Eaton P. (2004). Detection and mapping of widespread intermolecular protein disulfide formation during cardiac oxidative stress using proteomics with diagonal electrophoresis. J. Biol. Chem..

[27] Diering S., Stathopoulou K., Goetz M., Rathjens L., Harder S., Piasecki A., Raabe J., Schulz S., Brandt M., Pflaumenbaum J., Fuchs U., Donzelli S., Sadayappan S., Nikolaev V.O., Flenner F., Ehler E., Cuello F. (2020). Receptor-independent modulation of cAMP-dependent protein kinase and protein phosphatase signaling in cardiac myocytes by oxidizing agents. J. Biol. Chem..

[28] Ekhator E.S., Fazzari M., Newman R.H. (2025). Redox regulation of cAMP-Dependent protein kinase and its role in health and disease. Life.

[29] Simon J.N., Vrellaku B., Monterisi S., Chu S.M., Rawlings N., Lomas O., Marchal G.A., Waithe D., Syeda F., Gajendragadkar P.R., Jayaram R., Sayeed R., Channon K.M., Fabritz L., Swietach P., Zaccolo M., Eaton P., Casadei B. (2021). Oxidation of protein kinase A regulatory subunit PKARIalpha protects against myocardial ischemia-reperfusion injury by inhibiting lysosomal-triggered calcium release. Circulation.

[30] Trum M., Islam M.M.T., Lebek S., Baier M., Hegner P., Eaton P., Maier L.S., Wagner S. (2020). Inhibition of cardiac potassium currents by oxidation-activated protein kinase A contributes to early afterdepolarizations in the heart. Am. J. Physiol. Heart Circ. Physiol..

[31] Islam M.M.T., Tarnowski D., Zhang M., Trum M., Lebek S., Mustroph J., Daniel H., Moellencamp J., Pabel S., Sossalla S., El-Armouche A., Nikolaev V.O., Shah A.M., Eaton P., Maier L.S., Sag C.M., Wagner S. (2021). Enhanced heart failure in redox-dead Cys17Ser PKARIalpha Knock-In mice. J. Am. Heart Assoc..

[32] Shimokawa H. (2010). Hydrogen peroxide as an endothelium-derived hyperpolarizing factor. Pflügers Archiv.

[33] Prysyazhna O., Rudyk O., Eaton P. (2012). Single atom substitution in mouse protein kinase G eliminates oxidant sensing to cause hypertension. Nat. Med..

[34] Aramide Modupe Dosunmu-Ogunbi A., Galley J.C., Yuan S., Schmidt H.M., Wood K.C., Straub A.C. (2021). Redox switches controlling nitric oxide signaling in the resistance vasculature and implications for blood pressure regulation: mid-career award for research excellence 2020. Hypertension.

[35] Burgoyne J.R., Prysyazhna O., Rudyk O., Eaton P. (2012). cGMP-dependent activation of protein kinase G precludes disulfide activation: implications for blood pressure control. Hypertension.

[36] Vane J., Corin R.E. (2003). Prostacyclin: a vascular mediator. Eur. J. Vasc. Endovasc. Surg..

[37] Clapp L.H., Gurung R. (2015). The mechanistic basis of prostacyclin and its stable analogues in pulmonary arterial hypertension: role of membrane versus nuclear receptors. Prostag. Other Lipid Mediat..

[38] Rudyk O., Prysyazhna O., Eaton P. (2023). Oxidation of protein kinase a regulatory subunit PKARIα alpha regulates vasodilation and blood pressure lowering. Heart (British Cardiac Society).

[39] Burgoyne J.R., Rudyk O., Cho H.J., Prysyazhna O., Hathaway N., Weeks A., Evans R., Ng T., Schroder K., Brandes R.P., Shah A.M., Eaton P. (2015). Deficient angiogenesis in redox-dead Cys17Ser PKARI alpha knock-in mice. Nat. Commun..

[40] Byrne J.A., Grieve D.J., Bendall J.K., Li J.M., Gove C., Lambeth J.D., Cave A.C., Shah A.M. (2003). Contrasting roles of NADPH oxidase isoforms in pressure-overload versus angiotensin II-induced cardiac hypertrophy. Circ. Res..

[41] Trevelin S.C., Dos Santos C.X., Ferreira R.G., de Sa Lima L., Silva R.L., Scavone C., Curi R., Alves-Filho J.C., Cunha T.M., Roxo-Junior P., Cervi M.C., Laurindo F.R., Hothersall J.S., Cobb A.M., Zhang M., Ivetic A., Shah A.M., Lopes L.R., Cunha F.Q. (2016). Apocynin and Nox2 regulate NF-kappaB by modifying thioredoxin-1 redox-state. Sci. Rep..

[42] Zhang M., Brewer A.C., Schroder K., Santos C.X., Grieve D.J., Wang M., Anilkumar N., Yu B., Dong X., Walker S.J., Brandes R.P., Shah A.M. (2010). NADPH oxidase-4 mediates protection against chronic load-induced stress in mouse hearts by enhancing angiogenesis. Proc. Natl. Acad. Sci. U. S. A..

[43] Zhang M., Mongue-Din H., Martin D., Catibog N., Smyrnias I., Zhang X., Yu B., Wang M., Brandes R.P., Schroder K., Shah A.M. (2018). Both cardiomyocyte and endothelial cell Nox4 mediate protection against hemodynamic overload-induced remodelling. Cardiovasc. Res..

[44] Rudyk O., Prysyazhna O., Burgoyne J.R., Eaton P. (2012). Nitroglycerin fails to lower blood pressure in redox-dead Cys42Ser PKG1alpha knock-in mouse. Circulation.

[45] Rudyk O., Phinikaridou A., Prysyazhna O., Burgoyne J.R., Botnar R.M., Eaton P. (2013). Protein kinase G oxidation is a major cause of injury during sepsis. Proc. Natl. Acad. Sci. U. S. A..

[46] Christie I.N., Theparambil S.M., Braga A., Doronin M., Hosford P.S., Brazhe A., Mascarenhas A., Nizari S., Hadjihambi A., Wells J.A., Hobbs A., Semyanov A., Abramov A.Y., Angelova P.R., Gourine A.V. (2023). Astrocytes produce nitric oxide via nitrite reduction in mitochondria to regulate cerebral blood flow during brain hypoxia. Cell Rep..

[47] Rabbani A., Salehi S. (2017). Dynamic modeling of the formation damage and mud cake deposition using filtration theories coupled with SEM image processing. J. Nat. Gas Sci. Eng..

[48] Ezeakacha C.P., Rabbani A., Salehi S., Ghalambor A. (2018). SPE International Conference and Exhibition on Formation Damage Control.

[49] Rudyk O., Makra P., Jansen E., Shattock M.J., Poston L., Taylor P.D. (2011). Increased cardiovascular reactivity to acute stress and salt-loading in adult male offspring of fat fed non-obese rats. PLoS One.

[50] Boguslavskyi A., Tokar S., Prysyazhna O., Rudyk O., Sanchez-Tatay D., Lemmey H.A.L., Dora K.A., Garland C.J., Warren H.R., Doney A., Palmer C.N.A., Caulfield M.J., Vlachaki Walker J., Howie J., Fuller W., Shattock M.J. (2021). Phospholemman phosphorylation regulates vascular tone, blood pressure, and hypertension in mice and humans. Circulation.

[51] Rudyk O., Rowan A., Prysyazhna O., Krasemann S., Hartmann K., Zhang M., Shah A.M., Ruppert C., Weiss A., Schermuly R.T., Ida T., Akaike T., Zhao L., Eaton P. (2019). Oxidation of PKGIalpha mediates an endogenous adaptation to pulmonary hypertension. Proc. Natl. Acad. Sci. U. S. A..

[52] Goldstein D.S., Feuerstein G., Izzo J.L., Kopin I.J., Keiser H.R. (1981). Validity and reliability of liquid chromatography with electrochemical detection for measuring plasma levels of norepinephrine and epinephrine in man. Life Sci..

[53] Anton A.H., Sayre D.F. (1962). A study of the factors affecting the aluminum oxide-trihydroxyindole procedure for the analysis of catecholamines. J. Pharmacol. Exp. Therapeut..

[54] Wolhuter K., Whitwell H.J., Switzer C.H., Burgoyne J.R., Timms J.F., Eaton P. (2018). Evidence against stable protein S-Nitrosylation as a widespread mechanism of post-translational regulation. Mol. Cell.

[55] Intengan H.D., Schiffrin E.L. (2000). Structure and mechanical properties of resistance arteries in hypertension: role of adhesion molecules and extracellular matrix determinants. Hypertension.

[56] Burgoyne J.R., Eaton P. (2009). Transnitrosylating nitric oxide species directly activate type I protein kinase A, providing a novel adenylate cyclase-independent cross-talk to beta-adrenergic-like signaling. J. Biol. Chem..

[57] Smith K.A., Waypa G.B., Schumacker P.T. (2017). Redox signaling during hypoxia in mammalian cells. Redox Biol..

[58] Masi S., Uliana M., Virdis A. (2019). Angiotensin II and vascular damage in hypertension: role of oxidative stress and sympathetic activation. Vasc. Pharmacol..

[59] Bian J., Zhang S., Yi M., Yue M., Liu H. (2018). The mechanisms behind decreased internalization of angiotensin II type 1 receptor. Vasc. Pharmacol..

[60] Galley J.C., Straub A.C. (2017). Redox control of vascular function. Arterioscler. Thromb. Vasc. Biol..

[61] Stauss H.M. (2007). Identification of blood pressure control mechanisms by power spectral analysis. Clin. Exp. Pharmacol. Physiol..

[62] Thomas G.D. (2011). Neural control of the circulation. Adv. Physiol. Educ..

[63] Guimaraes S., Moura D. (2001). Vascular adrenoceptors: an update. Pharmacol. Rev..

[64] Zygmunt A., Stanczyk J. (2010). Methods of evaluation of autonomic nervous system function. Arch. Med. Sci..

[65] Yoshimoto M., Miki K., Fink G.D., King A., Osborn J.W. (2010). Chronic angiotensin II infusion causes differential responses in regional sympathetic nerve activity in rats. Hypertension.

[66] Goldsmith S.R., Hasking G.J. (1991). Effect of a pressor infusion of angiotensin II on sympathetic activity and heart rate in normal humans. Circ. Res..

[67] Katona M., Gladwin M.T., Straub A.C. (2023). Flipping off and on the redox switch in the microcirculation. Annu. Rev. Physiol..

[68] Bian K., Doursout M.F., Murad F. (2008). Vascular system: role of nitric oxide in cardiovascular diseases. J. Clin. Hypertens..

[69] Schlossmann J., Feil R., Hofmann F. (2003). Signaling through NO and cGMP-dependent protein kinases. Ann. Med..

[70] Mori A., Saito M., Sakamoto K., Nakahara T., Ishii K. (2007). Intravenously administered vasodilatory prostaglandins increase retinal and choroidal blood flow in rats. J. Pharmacol. Sci..

[71] Prysyazhna O., Eaton P. (2015). Redox regulation of cGMP-dependent protein kinase Ialpha in the cardiovascular system. Front. Pharmacol..

[72] Hofmann F. (2020). The cGMP system: components and function. Biol. Chem..

[73] Burgoyne J.R., Madhani M., Cuello F., Charles R.L., Brennan J.P., Schroder E., Browning D.D., Eaton P. (2007). Cysteine redox sensor in PKGIa enables oxidant-induced activation. Science.

[74] Muller P.M., Gnugge R., Dhayade S., Thunemann M., Krippeit-Drews P., Drews G., Feil R. (2012). H(2)O(2) lowers the cytosolic Ca(2)(+) concentration via activation of cGMP-dependent protein kinase Ialpha. Free Radic. Biol. Med..

[75] Majed B.H., Khalil R.A. (2012). Molecular mechanisms regulating the vascular prostacyclin pathways and their adaptation during pregnancy and in the newborn. Pharmacol. Rev..

[76] Sies H., Jones D.P. (2020). Reactive oxygen species (ROS) as pleiotropic physiological signalling agents. Nat. Rev. Mol. Cell Biol..

[77] Sies H., Belousov V.V., Chandel N.S., Davies M.J., Jones D.P., Mann G.E., Murphy M.P., Yamamoto M., Winterbourn C. (2022). Defining roles of specific reactive oxygen species (ROS) in cell biology and physiology. Nat. Rev. Mol. Cell Biol..

[78] Rudyk O., Eaton P. (2014). Biochemical methods for monitoring protein thiol redox states in biological systems. Redox Biol..

[79] Stubbert D., Prysyazhna O., Rudyk O., Scotcher J., Burgoyne J.R., Eaton P. (2014). Protein kinase G Ialpha oxidation paradoxically underlies blood pressure lowering by the reductant hydrogen sulfide. Hypertension.

[80] Kaszczewski P., Elwertowski M., Leszczynski J., Ostrowski T., Galazka Z. (2020). Volumetric carotid flow characteristics in doppler ultrasonography in healthy population over 65 years old. J. Clin. Med..

[81] Kelley E.E., Hock T., Khoo N.K., Richardson G.R., Johnson K.K., Powell P.C., Giles G.I., Agarwal A., Lancaster J.R., Tarpey M.M. (2006). Moderate hypoxia induces xanthine oxidoreductase activity in arterial endothelial cells. Free Radic. Biol. Med..

[82] Kelley E.E., Khoo N.K., Hundley N.J., Malik U.Z., Freeman B.A., Tarpey M.M. (2010). Hydrogen peroxide is the major oxidant product of xanthine oxidase. Free Radic. Biol. Med..

[83] Angelova P.R., Kasymov V., Christie I., Sheikhbahaei S., Turovsky E., Marina N., Korsak A., Zwicker J., Teschemacher A.G., Ackland G.L., Funk G.D., Kasparov S., Abramov A.Y., Gourine A.V. (2015). Functional oxygen sensitivity of astrocytes. J. Neurosci..

[84] Charles R.L., Schroder E., May G., Free P., Gaffney P.R., Wait R., Begum S., Heads R.J., Eaton P. (2007). Protein sulfenation as a redox sensor: proteomics studies using a novel biotinylated dimedone analogue. Mol. Cell. Proteomics.

[85] Knight H., Abis G., Kaur M., Green H.L.H., Krasemann S., Hartmann K., Lynham S., Clark J., Zhao L., Ruppert C., Weiss A., Schermuly R.T., Eaton P., Rudyk O. (2023). Cyclin D-CDK4 disulfide bond attenuates pulmonary vascular cell proliferation. Circ. Res..

[86] Loscalzo J. (2016). Adaptions to hypoxia and redox stress: essential concepts confounded by misleading terminology. Circ. Res..

[87] Pearce W.J. (1995). Mechanisms of hypoxic cerebral vasodilatation. Pharmacol. Ther..

[88] Mascarenhas A., Braga A., Majernikova S.M., Nizari S., Marletta D., Theparambil S.M., Aziz Q., Marina N., Gourine A.V. (2025). On the mechanisms of brain blood flow regulation during hypoxia. J. Physiol..

[89] Janssen B.J., Smits J.F. (2002). Autonomic control of blood pressure in mice: basic physiology and effects of genetic modification. Am. J. Physiol. Regul. Integr. Comp. Physiol..

[90] Wang Q., Hummler E., Maillard M., Nussberger J., Rossier B.C., Brunner H.R., Burnier M. (2001). Compensatory up-regulation of angiotensin II subtype 1 receptors in alpha ENaC knockout heterozygous mice. Kidney Int..

[91] Griendling K.K., Minieri C.A., Ollerenshaw J.D., Alexander R.W. (1994). Angiotensin II stimulates NADH and NADPH oxidase activity in cultured vascular smooth muscle cells. Circ. Res..

[92] Mehta P.K., Griendling K.K. (2007). Angiotensin II cell signaling: physiological and pathological effects in the cardiovascular system. Am. J. Physiol. Cell Physiol..

[93] Mollnau H., Wendt M., Szocs K., Lassegue B., Schulz E., Oelze M., Li H., Bodenschatz M., August M., Kleschyov A.L., Tsilimingas N., Walter U., Forstermann U., Meinertz T., Griendling K., Munzel T. (2002). Effects of angiotensin II infusion on the expression and function of NAD(P)H oxidase and components of nitric oxide/cGMP signaling. Circ. Res..

[94] Sowers J.R. (2002). Hypertension, angiotensin II, and oxidative stress. N. Engl. J. Med..

[95] Yayama K., Hiyoshi H., Imazu D., Okamoto H. (2006). Angiotensin II stimulates endothelial NO synthase phosphorylation in thoracic aorta of mice with abdominal aortic banding via type 2 receptor. Hypertension.

