# Supplementary material for: A redox sensor in protein kinase A regulatory subunit Iα regulates vasodilation and protects against hypertension
Source: Redox Biol. 2026 Mar 23;92:104136. doi: 10.1016/j.redox.2026.104136 (PMC13068537; doi:10.1016/j.redox.2026.104136)
Supplement: Supplementary file 1 — Supplementary Figure1. In vitro cell responses, ex vivo and in vivo vascular responses of wild-type or ‘redox-dead’ Cys17Ser PKARIα knock-in mice. A. Disulfide-PKARIα protein expression in thoracic aortae rings of WT mice subjected either to a vehicle or 50, 100, 200 and 500 μM H2O2 treatment for 30 min. B. Dose-dependent constriction of aortic rings from WT or PKA KI mice in response to U-46619. C. NECA-induced dose-dependent response of carotid arteries (pre-constricted with U-46619), from WT or PKA KI mice D. Internal circumference of left and right carotid common carotid arteries assessed by myography in WT and PKA KI mice. E. Basal carotid arterial flow parameters assessed by ultrasound Doppler in anesthetized WT or PKA KI mice. F. Basal cardiac function parameters assessed by ultrasound in anesthetized WT or PKA KI mice. ∗P< 0.05, ∗∗∗P< 0.001 versus vehicle or a respective WT. SMCs, smooth muscle cells; WT, wild type; PKA KI, Cys17Ser PKARIα knock-in mice; H2O2, hydrogen peroxide, acts a vasodilator; U-46619, stable synthetic analogue of the prostaglandin PGH2, acts as a thromboxane receptor agonist; NECA, 5′-N-Ethylcarboxamidoadenosine, non-selective adenosine receptor agonist. Supplementary Figure2. In vitro and ex vivo vascular responses to angiotensin II in wild-type or ‘redox-dead’ Cys17Ser PKARIα knock-in mice. A. Disulfide-PKARIα protein expression in aortic smooth muscle cells subjected to vehicle or AngII for 15 min (left). Disulfide-PKARIα protein expression in aortae rings of WT mice subjected either to vehicle, AngII or H2O2 for 5 min (right). B. Representative traces obtained by myography from WT or PKA KI mice in abdominal aortae or mesenteric arteries. Acute constriction of abdominal aortae rings in response to a single bolus dose of AngII in mid-aged WT or PKA KI mice (C), mid-aged Nox2-null mice (D) or mid-aged Nox4-null mice (E). ∗P< 0.05 versus vehicle or respective WT. M, PKARIα monomer; D, PKARIα dimer; H2O2, hydrogen peroxide; AngII, angi [file mmc1.pptx]

## Slide 1
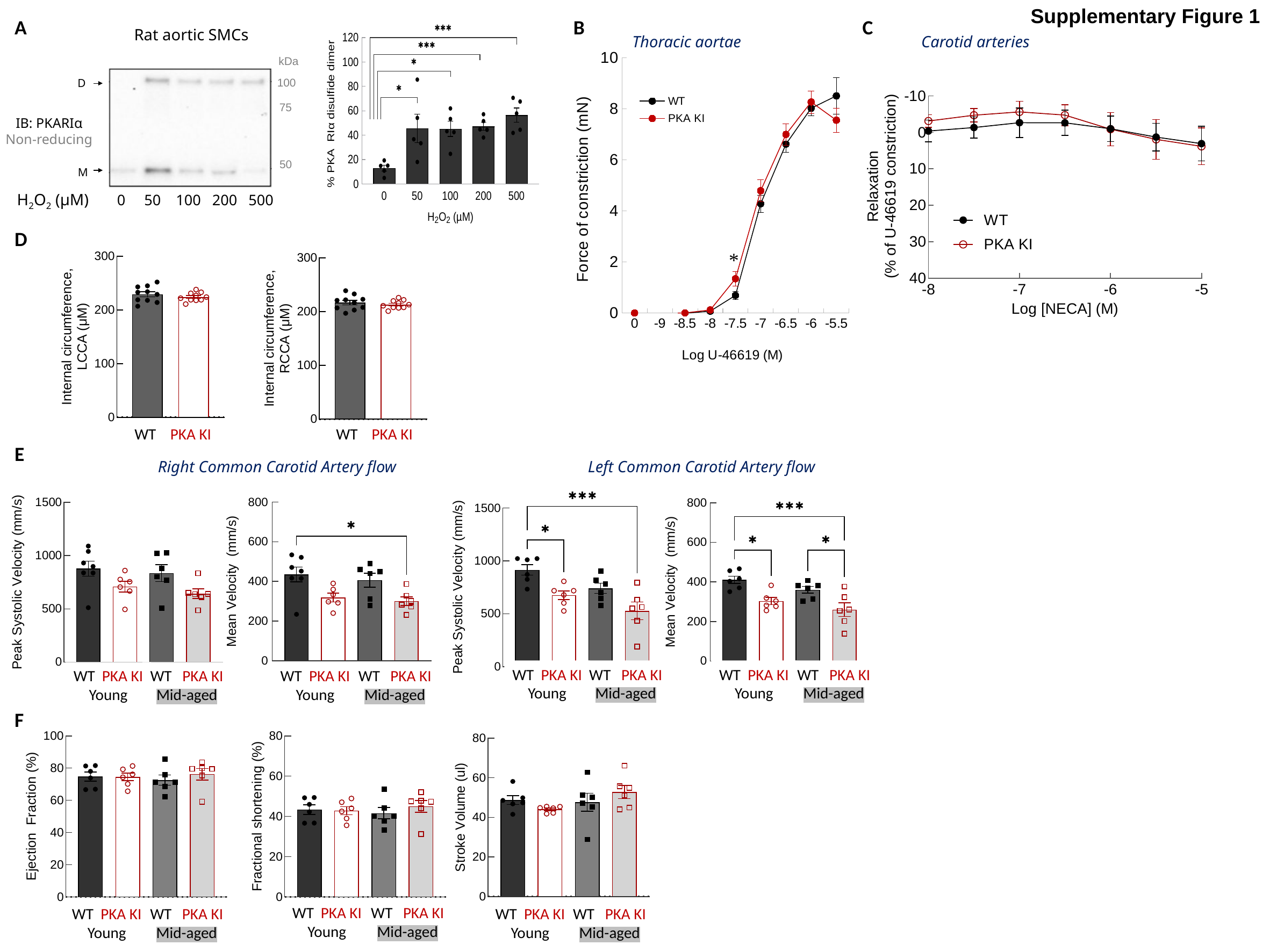

Supplementary Figure 1
A
B
C
Rat aortic SMCs
Thoracic aortae
Carotid arteries
### Chart
| Category | WT | PKA KI |
|---|---|---|
| 0 | 0.0 | 0.0 |
| -9 | -0.011966666666666632 | -0.006974999999999998 |
| -8.5 | -0.009566666666666588 | 0.0028300000000000326 |
| -8 | 0.0729055555555556 | 0.11984999999999997 |
| -7.5 | 0.6909055555555553 | 1.343 |
| -7 | 4.281172222222221 | 4.792655 |
| -6.5 | 6.6154111111111105 | 6.99812 |
| -6 | 8.023066666666667 | 8.26787 |
| -5.5 | 8.508275 | 7.554830000000001 |kDa
100
D
75
IB: PKARIα
Non-reducing
50
M
H2O2 (µM) 0 50 100 200 500
D
WT PKA KI
WT PKA KI
E
Right Common Carotid Artery flow
Left Common Carotid Artery flow
WT PKA KI
Young
WT PKA KI
Mid-aged
WT PKA KI
Young
WT PKA KI
Mid-aged
WT PKA KI
Young
WT PKA KI
Mid-aged
WT PKA KI
Young
WT PKA KI
Mid-aged
WT PKA KI
Mid-age
F
WT PKA KI
Young
WT PKA KI
Mid-aged
WT PKA KI
Young
WT PKA KI
Mid-aged
WT PKA KI
Young
WT PKA KI
Mid-aged

## Slide 2
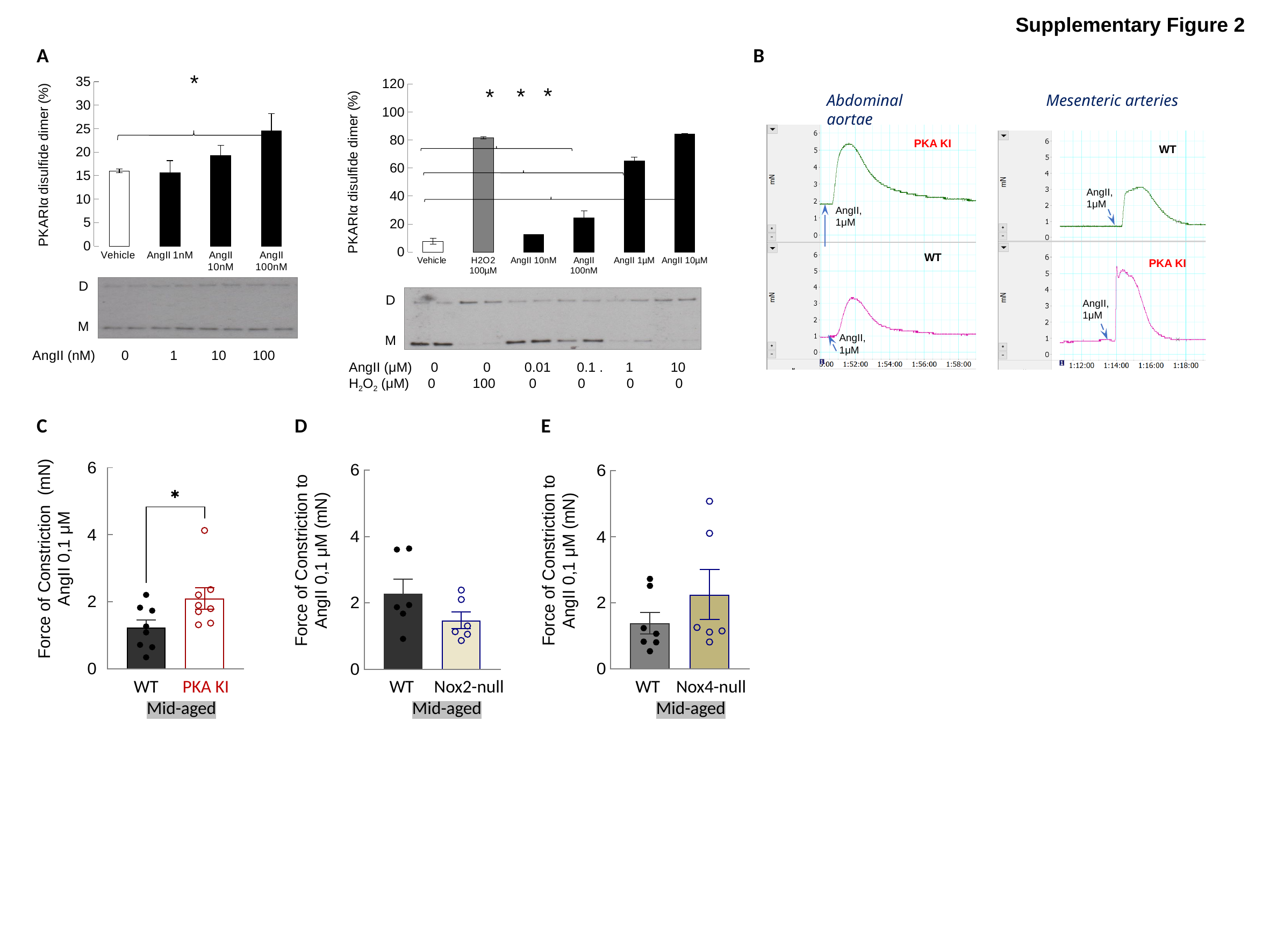

Supplementary Figure 2
A
B
### Chart
| Category | |
|---|---|
| Vehicle | 15.981315091242486 |
| AngII 1nM | 15.656571780426459 |
| AngII 10nM | 19.324110495493798 |
| AngII 100nM | 24.55758102838351 |
### Chart
| Category | |
|---|---|
| Vehicle | 7.714198428464725 |
| H2O2 100µM | 81.58938120116389 |
| AngII 10nM | 12.64662856301199 |
| AngII 100nM | 24.51001677155714 |
| AngII 1µM | 65.14959123120954 |
| AngII 10µM | 84.38120118161241 |Abdominal aortae
Mesenteric arteries
PKA KI
WT
AngII,
1μM
AngII,
1μM
WT
PKA KI
D
M
 AngII (nM) 0 1 10 100
D
M
AngII,
1μM
AngII,
1μM
 AngII (μM) 0 0 0.01 0.1 . 1 10
 H2O2 (μM) 0 100 0 0 0 0
C
D
E
WT PKA KI
Mid-aged
WT Nox2-null
Mid-aged
WT Nox4-null
Mid-aged

## Slide 3
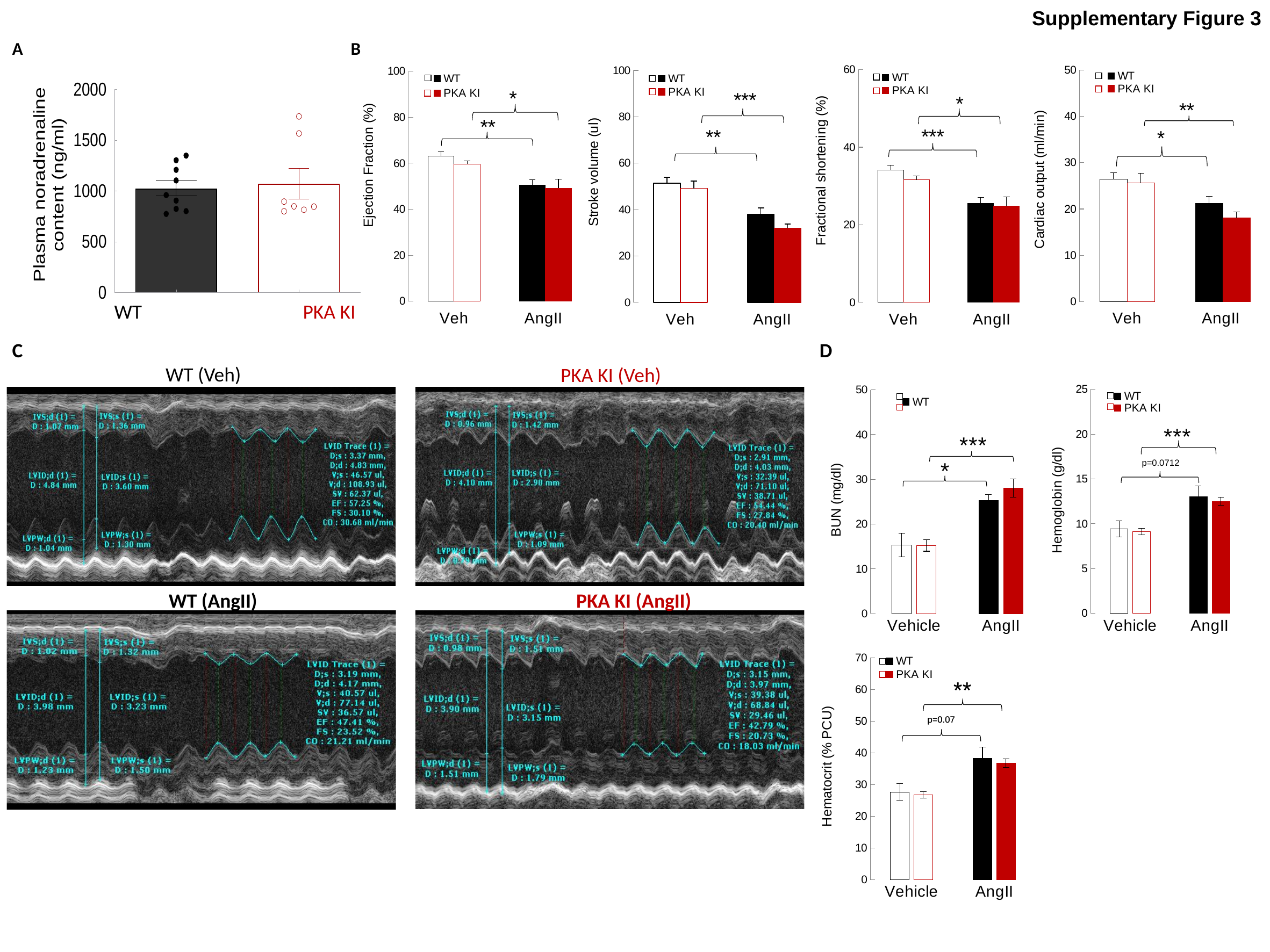

Supplementary Figure 3
### Chart
| Category | | |
|---|---|---|
| Veh | 63.186190476190475 | 59.67041666666667 |
| AngII | 50.402249999999995 | 49.108333333333334 |
A
B
### Chart
| Category | | |
|---|---|---|
| Veh | 34.05547619047619 | 31.577500000000004 |
| AngII | 25.551166666666667 | 24.775952380952383 |
### Chart
| Category | | |
|---|---|---|
| Veh | 26.42642857142857 | 25.61447916666667 |
| AngII | 21.247999999999998 | 18.076190476190472 |
### Chart
| Category | | |
|---|---|---|
| Veh | 51.32880952380953 | 49.165625000000006 |
| AngII | 37.875416666666666 | 31.893809523809523 |
WT 		 PKA KI
C
D
WT (Veh)
PKA KI (Veh)
WT (AngII)
PKA KI (AngII)
### Chart
| Category | WT | PKA KI |
|---|---|---|
| Vehicle | 9.4 | 9.1 |
| AngII | 13.0 | 12.5 |
### Chart
| Category | WT | PKA KI |
|---|---|---|
| Vehicle | 15.333333333333334 | 15.25 |
| AngII | 25.25 | 28.0 |
### Chart
| Category | WT | PKA KI |
|---|---|---|
| Vehicle | 27.666666666666668 | 26.75 |
| AngII | 38.25 | 36.75 |
